# Prospects of Polymeric Nanocomposite Membranes for Water Purification and Scalability and their Health and Environmental Impacts: A Review

**DOI:** 10.3390/nano12203637

**Published:** 2022-10-17

**Authors:** Nouf F. Al Harby, Mervette El-Batouti, Mahmoud M. Elewa

**Affiliations:** 1 Department of Chemistry, College of Science, Qassim University, Qassim 52571, Saudi Arabia; 2 Chemistry Department, Faculty of Science, Alexandria University, Alexandria 21526, Egypt; 3 Arab Academy for Science, Technology and Maritime Transport, Alexandria P.O. Box 1029, Egypt

**Keywords:** polymeric nanocomposite membranes, nanoparticles, located polymerisation, physical combining, sol-gel, electrospinning, 3D printing, size exclusion mechanism, dissolution–diffusion mechanism, stability, scalability, persistence and toxicity

## Abstract

Water shortage is a major worldwide issue. Filtration using genuine polymeric membranes demonstrates excellent pollutant separation capabilities; however, polymeric membranes have restricted uses. Nanocomposite membranes, which are produced by integrating nanofillers into polymeric membrane matrices, may increase filtration. Carbon-based nanoparticles and metal/metal oxide nanoparticles have received the greatest attention. We evaluate the antifouling and permeability performance of nanocomposite membranes and their physical and chemical characteristics and compare nanocomposite membranes to bare membranes. Because of the antibacterial characteristics of nanoparticles and the decreased roughness of the membrane, nanocomposite membranes often have greater antifouling properties. They also have better permeability because of the increased porosity and narrower pore size distribution caused by nanofillers. The concentration of nanofillers affects membrane performance, and the appropriate concentration is determined by both the nanoparticles’ characteristics and the membrane’s composition. Higher nanofiller concentrations than the recommended value result in deficient performance owing to nanoparticle aggregation. Despite substantial studies into nanocomposite membrane manufacturing, most past efforts have been restricted to the laboratory scale, and the long-term membrane durability after nanofiller leakage has not been thoroughly examined.

## 1. Introduction

The world’s rapidly expanding population places enormous strain on clean water supplies. According to the World Health Organization, by 2025, half of the world’s population will live in water-stressed regions [1]. It is critical to protect existing freshwater sources and create technology to supplement the present water supply [2]. Industrial waste, municipal wastewater, and agricultural operations are major contributors to water contamination. Pesticides [3], hazardous heavy metals [4], organic acids [5], fertilisers [6], dyes [7], and microbes [8] are all found in wastewater. Heavy metals are the most dangerous of the above-mentioned contaminants owing to their toxicity and non-biodegradability [9,10,11]. Therefore, they cling to the food chain and the environment [12]. Furthermore, drinking dirty water causes a variety of ailments, including cancer, nasal septum rupture, skin ulcers and irritation, organ damage, diarrhoea, fever, chills, headache, stomach discomfort, appetite loss, and many others [13]. As a result, removing these contaminants is critical to ensuring the availability of clean water for all living species [13].

Researchers have focused on membranes as the central component of membrane-based processes for a wide range of water treatment purposes, including the removal of harmful and non-harmful chemicals, heavy metals, and biological contaminants [14]. Membrane filtration is regarded as the most promising technique for addressing contemporary water concerns since it needs no chemical or heat input and produces no toxic by-products [9,15]. More crucially, by altering membrane architectures and pore sizes, membrane filtration may selectively remove pollutants [14,16]. Based on membrane pore diameters and working processes, membrane science and technology have resulted in the creation of several approaches, including microfiltration (MF) [17,18,19], ultrafiltration (UF) [20,21,22], nanofiltration (NF) [23,24,25], reverse osmosis (RO) [26,27,28], pervaporation (PV) [29,30,31], membrane distillation (MD) [32,33,34], and forward osmosis (FO) [35,36,37]. While MF is usually used for the removal of viruses, colloids, and macromolecules, UF is frequently utilised for the removal of suspended particles, prokaryotes, yeasts, and fungi. Nanofiltration mostly removes hardness, heavy metals, and dissolved organic matter, while RO, PV, and MD are used for desalination, water reuse, and the production of ultrapure water. [2,38,39].

Membranes are classified into polymeric membranes and inorganic membranes based on their composition. Both membrane types have been widely researched for water filtration. Inorganic membranes are very resistant to corrosive agents such as strong acids, bases, and oxidants, as well as have superior mechanical strength and temperature tolerance. Inorganic membranes outperform polymeric membranes in terms of maintenance because they are less susceptible to bacterial deterioration. High-temperature chemical cleaning may be utilised to remove biofouling and obtain high flux recovery. Inorganic membranes are the sole solution in many applications that demand extreme environmental conditions. Inorganic membranes, on the other hand, are less frequent in water treatment owing to their high production costs, handling challenges, and relatively poor control over pore size distribution [40,41]. Polymeric membranes, on the other hand, are very adaptable. Their pore diameters may be narrowed to a certain range. By altering the casting circumstances, additives, coagulation bath conditions, and monomer molecules and concentrations, the membrane characteristics may be altered [40]. However, polymeric membranes have several significant limitations. One major downside is that they are prone to clogging owing to their intrinsic hydrophobicity [42,43,44]. They are also susceptible to chlorine. Depending on the level of fouling, significant physical and chemical cleaning or membrane replacement may be necessary [41,45]. As a frequent disinfectant in the water treatment process, chlorine may react with electron-rich functional groups in polymeric membranes [46]. Another difficulty is the substantial trade-off between permeability and selectivity. For the existing polymeric membrane, it is impossible to enhance one without sacrificing the other.

**Figure 1:**
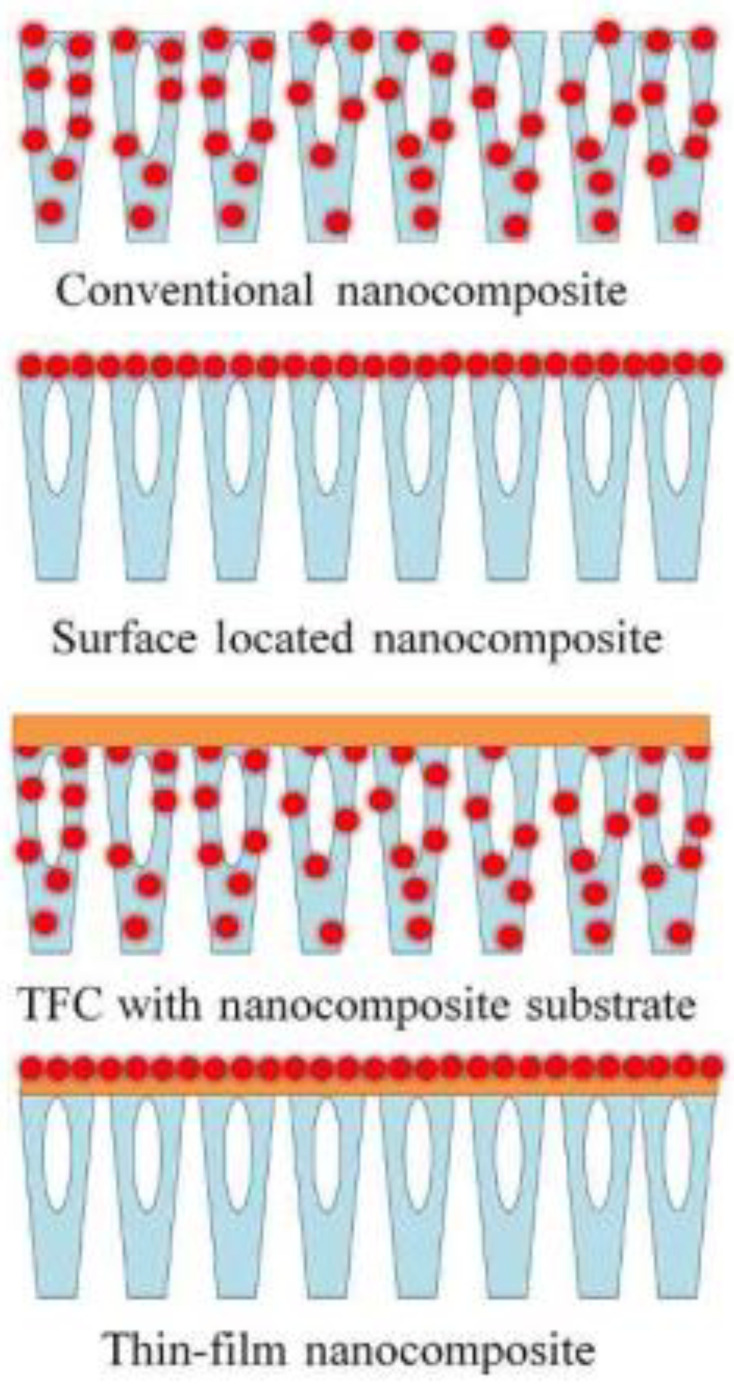
A diagram depicting the forms of nanocomposite membranes.

Due to the limits of present polymeric membranes, next-generation membranes with excellent permeability and selectivity, as well as antifouling and chlorine resistance, have been developed. Nanotechnology advancements, an enabling technology at the atomic level, give a once-in-a-lifetime opportunity for membrane development. Engineered nanoparticles having well-controlled characteristics in at least two dimensions in the range of 1–100 nm may be readily manufactured. Because the quantum size effect becomes substantial at the nanoscale, they have distinct structural, thermal, and mechanical characteristics as compared to their bulk counterparts. Their distinct features are also due to their greatly enhanced specific surface area [47]. The drive to include nanoparticles in polymeric membranes stems from the belief that polymeric membranes may benefit from the better features of nanoparticles to overcome some of their shortcomings, such as fouling proclivity. As shown in Figure 1, depending on the membrane structure and placement of the nanoparticles, many kinds of nanocomposite membranes may be made, including traditional mix-matrix nanocomposite (MMN), thin-film nanocomposite (TFN), thin-film composite (TFC) with nanocomposite substrate, and surface-located nanocomposite. [14,16]. Organic polymers, primarily polysulfone (PSF) [48,49,50,51,52], poly(ether sulfone) (PES) [53], polyacrylonitrile (PAN) [54,55], polyamide [56,57], polyimide [58,59], cellulose acetate membrane [60,61,62,63], polylactic acid [64,65,66,67], polyvinyl alcohol (PVA) [68,69,70], poly-(vinylidene fluoride) (PVDF) [71,72,73,74], and polytetrafluoroethylene (PTFE) [75,76,77], are all often employed in water filtering membranes.

This research gives an in-depth look at several production methods for nanocomposite polymeric membranes by traditional methods reaching 3D Printing Innovation with unique functionalities provided by diverse nanomaterials and their prospective water treatment applications. To that end, a picture of unrealised potential and difficulties with present-day membrane systems is provided. The complete assessment of the scalability of such membranes is also explored regarding health, environmental, and cost-efficient impacts, as well as the research required to solve these challenges. Recent advances in the synthesis of innovative nanostructure materials have also addressed the possibility of creating new hybrid membranes for water treatment applications. Several significant obstacles in nanocomposite membrane creation are explored at the conclusion of the paper, as are future research requirements.

## 2. Nano-Enhanced and Nanostructured Membranes

There is a significant distinction between nano-enhanced and nanostructured membranes in membrane technology. The phrase “nano-enhanced” refers to membranes that have been functionalised with nanoparticles, nanotubes, nanosheets, or nanofibers, while the word “nanostructured” refers exclusively to the pore size or internal structure of the membrane or the size of the solutes separated by the membrane (approximately 1 nm).

Despite the tremendous success and widespread use of nanostructured membranes (e.g., nanofiltration (NF) membranes), numerous limitations remain, including fouling, restricted flux, and the trade-off between flow and selectivity. To circumvent these constraints, nanoparticles may be introduced into the membrane matrix to enhance membrane properties such as antifouling and antibacterial properties, selectivity, and flow (through altering the membrane hydrophilicity). The incorporation of nanoparticles into the membrane may help reduce energy usage, the use of chemicals for membrane cleaning, and overall operating costs [78]. The degree of technological advancement and the danger of using nano-enhanced and nanostructured membranes vary greatly. The major membrane technology companies are still not engaged in large-scale manufacturing of nano-enhanced membranes [79].

## 3. Nanoparticles

As the size of solid particles decreases to around 1/1,000,000 of a millimetre, the number of atoms comprising a particle decreases to a few hundred or thousands. When this stage is achieved, the particle’s basic physical characteristics, such as its melting point, may be drastically changed; ceramic material may sinter at lower temperatures than before. In addition, when particles become smaller than the wavelength of visible light, they become transparent and emit a unique kind of light through plasma absorption. They exhibit completely different electromagnetic and/or physiochemical properties than when they exist in bulk, although being formed of the same materials. Nanoparticles are characterised variously based on their composition and the sectors and applications they are utilised in. Nanoparticles are defined as particles smaller than 10–20 nm, which is the size range at which the physical properties of solids are significantly influenced. However, nanoparticles are particles with a size range in the triple digits of nanometres (1 nm to 1 µm). Much study has been conducted in recent years on the use of nanoparticles in the synthesis and design of novel materials. Numerous scientific and technical disciplines have shown interest in these materials because of the exceptional differences in their mechanical, thermal, and magnetic characteristics compared to organic polymers [80,81,82,83].

Several inorganic nanoparticles have been shown to be harmful to certain bacteria [84]. This feature may be helpful for drinking water disinfection and antibacterial applications. Among organic nanoparticles, Ag nanoparticles are the most intriguing due to their very potent antibacterial and antifungal activities. In addition to being extremely selective towards certain microbes, Ag nanoparticles (Ag NPs) are also tiny enough to readily enter biological entities. Numerous hypotheses [85,86,87] have been advanced to explain how the mechanism producing antibacterial action works. Two primary processes account for the toxicity and antibacterial activity of Ag NPs [87,88]: the production of oxidative stress cells and reactive oxygen species. In the presence of Ag NPs, the cell membrane ruptures during endocytosis and in other locations, enabling ions and/or nanoparticles to enter the cell. Significant advances have been made in the development of water treatment membranes containing Ag nanoparticles produced on-site, and some commercial applications have been introduced. Reducing Ag NPs in situ using a moderate reducing agent (gaseous CO) over a polyacrylonitrile (PAN) substrate to promote biofilm resistance, so facilitating the production of an Ag-based thin-film composite (TFC) membrane, is a recent innovation [89].

Fe-based membranes are better in iron suspensions due to the fact that membranes prevent particle aggregation during the particle creation process, hence managing environmental dissolution issues during a chemical reaction; this also provides particle size control [90].

Similarly, Fe nanoparticles produced in situ and coated with Pd nanoparticles were used to functionalise polyvinyl pyrrolidone (PVP) decorated (hydrophilization) hollow fibre and flat sheet membranes for the elimination of chloro-organic hazardous substances, as palladium demonstrated full dechlorination without intermediates [91].

Using commercial PVDF membranes functionalised with polyacrylic acid (PAA) that included Fe/Ni bimetallic nanoparticles [92], an experiment was conducted to investigate the degradation of methyl orange dye. Polymerisation in situ resulted in a more uniform dispersion of metal without agglomeration on membranes than the dip-coating approach of PAA immobilisation was able to achieve.

By performing polymerisation in situ, PAA functionalisation resulted in a more uniform distribution of metal without agglomeration on membranes than could be achieved by the dip-coating approach of PAA immobilisation. Moreover, the research demonstrated that employing a nanocomposite membrane enhanced with in situ PAA polymerisation resulted in metal oxide nanoparticles being favourable because they have a simple and well-developed technique of production, they improve the hydrophilicity of the membrane, and they are cost-effective. Because TiO_2_ has remarkable chemical and physical properties, it is expected to be beneficial for water filtering. Numerous studies are being conducted on the use of in situ produced TiO_2_ nanoparticles in water treatment membranes. This level of interest is primarily due to two factors: firstly, its excellent photocatalytic activity and photo-induced ultra-hydrophilicity, both of which either repel or degrade organic foulants, thereby enhancing antifouling mechanisms; and secondly, its straightforward synthesis strategy, which allows it to be easily incorporated into the membrane fabrication process. The standard method for preparing TiO_2_-coated membranes in situ is to control the synchronous formation of TiO_2_ nanoparticles alongside a polymeric membrane as part of the non-solvent-induced phase separation (NIPS) method using the sol-gel technique, which fixes these newly constructed nanoparticles within the matrix of the membrane. Notably, careful control of the reaction rate and morphologies of nanoparticles requires a harsh microenvironment (containing acid or basic catalyst), which restricts their general use [92,93]. In imitation of natural biomaterials, biomimetic mineralisation has facilitated the creation of complex-structured metal-oxide-nanoparticle-based membranes in recent years. To perform this, nanoparticles of metal oxide with a hierarchical form, tenable size, and controlled dispersion are synthesised in situ in an aqueous solution at ambient temperature and neutral pH using an organic matrix. Organic matrices act as confined compartments and structural templates, as well as being important in the nucleation/growth of inorganic particles [94,95]. These nanocomposite membranes do not experience favourable excess aggregation or severe circumstances during the sol-gel process because biomimetic mineralisation occurs inside organic matrices, and the polymers and inorganic particles interface well. A unique TiO_2_-based membrane for water treatment was created by combining biomimetic mineralisation with the NIPS oil/water separation technology. The superoleophobicity of hybrid membranes was enhanced by a quick mineralisation process in situ using titanium (IV) bis (ammonium lactate) dihydroxide as a precursor and PVDF-g-poly 2-(methacryloyloxy) ethyl trimethylammonium chloride (PTA) as a surfactant. In the process of separating oil-in-water emulsions, membranes exhibited little flux decrease and practically total flux recovery, demonstrating a significant increase in oil–water separation performance as well as enhanced antifouling capabilities. Photoactive MF membranes using the controlled production of TiO_2_ nanoparticles on a wet polymer surface with tetraisopropoxide (TTIP) as a precursor were used to remove drugs from water. When tested with ibuprofen and diclofenac, membranes coated with hydrophilic TiO_2_ exhibited remarkable photocatalytic activity, and they have been acknowledged as an effective method for eliminating such medicines from water. Deposition of a polydopamine/polyethyleneimine (PDA/PEI) intermediate layer on the membrane’s surface offers intriguing potential for in situ modification utilising TiO_2_ nanoparticles to increase the membrane’s surface stability. Additionally, SiO_2_ nanoparticles have been successfully used to decorate novel water treatment membranes. By combining the in situ preparation process with the production of SiO_2_ nanoparticles, it is possible to develop a platform for the further enhancement of membrane performance. Researchers are comparing the effects of in situ and ex situ created SiO_2_ nanoparticles on the structural and performance characteristics of cellulose acetate membranes [96,97]. Controlling the condensation and hydrolysis of tetraethoxysilane (TEOS) inside a PES polymer matrix to initiate the in situ synthesis of SiO_2_ nanoparticles is considered the classic application of the in situ preparation technology. A recent study changed polyvinyl chloride (PVC) hollow fibre membranes using the soft hydrolysis reaction of TEOS in water utilising an environmentally friendly chemical technique, an in situ sol-gel procedure [98]. This approach does not need corrosive ingredients, either acid or alkali, as part of the coagulation liquid, hence reducing the cost of membrane production. For enzyme separation, further study described the in situ synthesis of amino-functionalised SiO_2_ nanoparticles in a poly (ether imide) matrix. Using an ion-exchange approach, homogenous ZrO_2_ nanoparticles in a polymeric matrix have been successfully synthesised. These in situ generated ZrO_2_ nanoparticles battle hydrophilicity and reduce protein adsorption in the membranes formed. This method efficiently eliminated both brittleness and shrinkage, two issues that are constantly faced when working with stiff materials. The mineral coating provides the membranes with exceptional curl resistance and mechanical stability, allowing them to withstand water treatment pressure filtration. Controlling the hydrolysis of Zr(SO_4_)_2_ on a PDA/PEI-coated UF membrane resulted in the formation of a defect-free ultrathin (10–20 nm) and strong ZrO_2_ film. Due to the ultrathin and hydrophilic nature of this inorganic selective layer, the membrane produced had a high water flow (60 Lm^−2^ under 0.6 MPa) and retention (>90 percent) for bivalent cations. It was shown that the coordinating force of the coating and the nanoparticles produced in situ was long-lasting. Comparable to nanoparticles based on similar metal oxides, Al-based nanoparticles contain characteristics that may result in improved membrane function. However, the use of in situ formed Al species in water treatment contexts has not yet been thoroughly studied. It has been shown that combining the sol-gel approach with the NIPS method provides an in situ preparation strategy for producing Al-containing PVDF UF membranes with superior separation performance. The surface of PVDF membranes was effectively changed by doping them with anhydrous and hydrated Al_2_O_3_ particles by in situ particle embedding, followed by crystal formation in a hydrothermal environment. Particles were entrapped on the surface of the PVDF membrane during the polymerisation after being dispersed in the membrane precipitation water bath. Using embedded Al_2_O_3_ particles as “roots”, the mineral phase on the surface of a membrane may be changed by growing hydrated Al_2_O_3_ using Al_2_(SO_4_)_3_ as a precursor. Embedded Al_2_O_3_ nanoparticles were placed on membranes in order to improve their anti-biofouling characteristics [99] due to the membranes’ unique qualities. This in situ preparation method for synthesising non-metal/metal oxide nanomaterials is expanding the range of applications for the next generation of nano-enhanced membranes. As a result of their large surface area and porous structure, porous inorganic solids have gained a great deal of attention among the available nanomaterials due to their ability to increase both the membrane’s overall porosity and its separation capacity. Based on this technology, several inorganic nanomaterials, such as β-FeOOH nanorods [99], CaCO_3_ nanoparticles [100], and Mg nanoparticles, are produced. Two (OH) nanoparticles have also been put into water treatment membranes. In addition to inorganic nanoparticles, polymeric nanoparticles have the potential to generate new materials with novel structure/property correlations.

## 4. Membranes Made of Polymeric Nanocomposite (PNC)

PNCs may be classified into three categories based on the functional properties of the membranes. Type I is conventional PNCs, which are PNCs created from polymeric nanocomposites to increase their characteristics but without adding a function to the membrane. The membrane is enhanced in this scenario, but it remains a passive element in separation systems, with the primary and unique role of separating components of the feed phase through two different mechanisms, ion exchange and ionic exchange, in addition to size exclusion or dissolution–diffusion [101,102,103,104,105,106,107]. Type II is the active-bulk phase PNCs, which are PNCs made from polymeric nanocomposites that give the membrane the ability to perform dual functions, one major function linked to mass transfer between two separated phases and a second function linked to specific properties of the material from which it is made; an example of this is a PNC made from inorganic nanoparticles distributed in a polymeric phase based on a conductive polymer such as poly(aniline) [108,109,110,111,112]. Type III is the active-surface PNC, which exhibits a second functionality on the surface in contact with the feed phase or the inner of the pores, but not in the bulk of the material [113,114,115,116,117,118]. The kinds of PNCs are shown in Table 1. The categorisation previously given is implicitly dependent on the nature of the active layer; hence, the same membrane might be classed as two distinct categories due to the effect of polymer bulk characteristics on polymeric surface properties.

Several papers have proposed a second category for the different kinds of PNCs based on the membrane structure and placement of nanomaterials: (i) mix-matrix PNCs, which are membranes made of some nanocomposite material, (ii) nanomaterial thin-film PNCs, which are membranes surface coated with some sort of nanomaterial, (iii) thin-film PNCs with nanocomposite substrate, which are membranes made of a thin-film on a support made of nanocomposite material, (iv) nanocomposite thin-film PNCs, which are membranes coated with some type of polymer nanomaterial [119].

Despite the fact that this classification aids in visualising the configuration of membrane layers, it is deemed unsuitable for describing the structure–function relationship of PNCs because all separation properties of porous membranes are dependent on the “active layer”, which would be defined as the layer with the littlest pore size distribution constituting the membrane structure, and as a result, the porous substrate must be understood to explain the separation properties. An asymmetric polymeric membrane, for example, is defined as porous polymeric support covered on its surface by a thin film with a pore-size distribution less than the support’s pore-size distribution; as a result, all retention characterisations in this type of membrane are largely decided by the thin film comprising the active layer, as the porous polymeric support’s function is to enhance the mechanical characteristics of the multi-layered system [120]. In the cases of “nanomaterial thin-film PNC” (symmetric membrane|nanomaterial), “thin-film PNC with nanocomposite substrate” (nanocomposite support|active layer), and “surface-coated PNC” (support|activelayer|nanomaterial), the previous classification describes multilayer membranes rather than PNCs. Note that the vertical bar was utilised to denote the contact between the membrane’s distinct layers. Note that “mix-matrix PNC” corresponds to symmetric PNC whereas “thin-film PNC” relates to asymmetric PNC [121].

## 5. Methods of Membrane Preparation

Hybrid inorganic polymer membranes or polymer nanocomposite membranes (PNCs) are cost-effective, high-performance membranes in which it is amazingly simple to manufacture and disseminate a wide variety of inorganic nanomaterials in the organic matrix. In this section, the primary approaches for producing hybrid inorganic/polymer membranes are outlined. Following the presentation of traditional approaches are innovative preparation techniques, such as electrospinning and 3D printing.

### 5.1. Traditional Methods for the Preparation of Nanocomposite Membranes

Currently, the conventional techniques for fabricating hybrid nanocomposite membranes fall into three categories. The methods include (1) in situ polymerisation, (2) sol-gel, and (3) physical mixing. These three methods may be utilised alone or in combination to create membrane architectures of choice [122,123,124].

#### 5.1.1. Located Polymerisation

**Figure 2:**
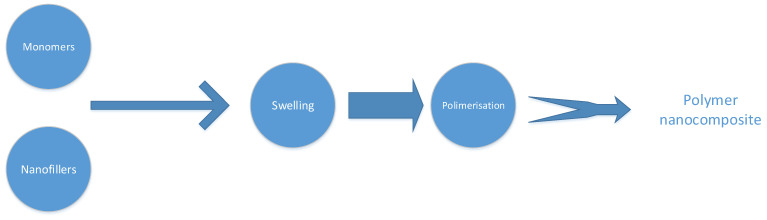
Steps for PNC fabrication using in situ polymerisation.

Infiltration, often known as in situ polymerisation, is a typical approach for producing PNC membranes. Before polymerisation, the nanofillers are mixed in bulk or solution with the monomer (Figure 2). When exposed to heating, high-energy radiation, or plasma, certain functional groups on the surface of nanofillers, such as hydroxyl and carboxyl groups, may create activated species (radicals, cations, or anions). These activated species may cause the monomer’s surface to polymerise. The polymer chain grows after the initiation phase, and inorganic fillers may be physically or chemically connected to or integrated into the polymer matrix. This method is particularly useful for nanocomposite membranes with inorganic exfoliated structures because the monomer penetrates the exfoliated structure’s inorganic galleries and, after polymerisation, achieves more uniform filler dispersion within the polymer matrix. Ring-opening, atom transfer radical, live anionic/cationic, and nitroxide-mediated polymerisations, in addition to standard polymerisation procedures [125,126,127], are among the polymerisations that may be employed in this methodology. The aggregation of inorganic nanofillers in the produced membranes is difficult to prevent or minimise using this approach [124,128,129,130,131].

As previously stated, many polymerisation techniques may be utilised during this preparation procedure, such as polystyrene (PS), which typically polymerises by generating radicals. This kind of polymerisation may occur in bulk, solution, or suspension. For this reason, the selection of the proper surfactant must take into consideration several criteria. The first is because the surfactant alters the reactivity of the inorganic filler so that it may react with the monomer. The second theory depends on the notion that a surfactant’s structure must have long alkyl chains or tetrahedral structures for interlayer space to be increased [132]. A team from the Toyota Research Institute was the first to use in situ polymerisation to create nylon-6/clay nanocomposite membranes. Between the layers, polymerisation occurs, and macromolecular chains are created. As these chains expand, they remove the disoriented clay layers, resulting in an exfoliated structure [133].

Doucouré et al. [134] created certain gas permeation membranes by using in situ plasma polymerisation on mesoporous silica containing various fluorinated monomers. The monomers used were octafluorocyclobutane (C_4_F_8_), trifluoromethane (CHF_3_), and 1,1,1,2-tetrafluoroethane (CF_3_-CH_2_F), with argon serving as the carrier gas. The Fourier transform infrared (FTIR) spectra and X-ray photoelectron spectroscopy (XPS) findings revealed that CF_3_-CH_2_F produces the most crosslinked polymers and C_4_F_8_ produces the least crosslinked polymers, indicating that C_4_F_8_ is the most flexible polymer. Using permeability and selectivity procedures, it was determined that N_2_ molecules could pass through this polymer with the least amount of crosslinking. Using CF_3_-CH_2_F, however, it proved difficult to penetrate tiny atoms such as He.

Patel et al. [135,136] produced various hybrid membranes by combining dispersed silica nanofillers with diacrylate-terminated poly(ethylene glycol) (PEG) or poly(propylene glycol) (PPG) and initiating the radical polymerisation using 2,2-azobisisobutyronitrile (AIBN). After silica incorporation, the polymer’s elastic modulus increased and its gas permeability decreased, but the CO_2_/H_2_ selectivity was practically unaffected. Moreover, Nunes et al. [137] produced a PNC for gas separation by dispersing silica in poly (ether imide) (PEI). In situ hydrolysis and condensation of tetraethoxysilane (TEOS) were used to produce polymer nanocomposite membranes to scatter silica, and aminosilanes, which interact strongly with the imide groups of PEI, were added into this inorganic polymer network. By measuring the glass transition temperature (Tg), it was discovered that aminosilanes raised the Tg, which is directly connected to the increase in rigidity; this rigidity explains why a PNC is more stable under pressure. In addition, the inclusion of silica transformed the membrane’s structure from finger-like to sponge-like [138].

#### 5.1.2. Sol-Gel

**Figure 3:**
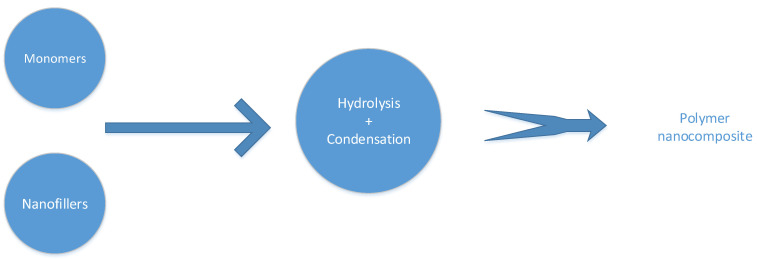
Steps to fabricate PNC with the sol-gel method.

The sol-gel method is a low-temperature synthesis technique that has been widely used to produce PNCs since the 1980s. This approach involves the mixing of dissolved inorganic nanofillers with monomers, oligomers, or polymers. Then, the inorganic precursors hydrolyse and condense into nanofillers that are uniformly scattered in the polymer matrix (Figure 3). The mid-reaction circumstances in this approach, such as room temperature and pressure, are advantageous. Additionally, the concentration of the various species in the solution is simple to regulate. Due to the dispersion of chemicals at the molecular or nanometre level [138,139,140,141,142,143], the membranes formed are homogenous.

Clearly, the actual process is more complicated, and intermediate compounds, such as metal oxo alkoxides, may be produced. To produce monodisperse TiO_2_ powder, for instance, hydrolysis, condensation, nucleation, and particle growth are undertaken in sequence. Oxo oligomers, polymers, and crosslinked macromolecules are also produced and coexist with the sol [144]. Typically, monomers and oligomers in gels condense and reprecipitate, resulting in a phase change. Some inorganic fillers are not overly sensitive to hydrolysis, and if water is introduced within a few days, gelation may occur. Therefore, hydrolysis and condensation happen without catalysts for non-silicate metal alkoxides [145], whereas acid or base catalysts are needed for silicon alkoxides.

Iwata et al. [146] produced gas-permeable PNC membranes to separate N_2_ and O_2_ by combining tetraethoxysilane (TEOS) with 1.3 to 20% fumed silica in polyacrylonitrile (PAN) membranes. Si–O–Si networks are created using TEOS. The scientists exhibited an excellent separation of the O_2_/N_2_ mixture by increasing the silica content of this PNC, thus producing a thick Si–O–Si network. In another instance, Gomes et al. [147] used sol-gel copolymerisation of TEOS with various organoalkoxysilanes to create a poly(1-trimethylsilyl-1-propyl) (PTMSP)/silica PNC. The kind and content of organoalkoxysilanes, as well as the temperature and duration of the synthesis process and gas permeability of the PNC, were examined. Butane permeability and butane/methane selectivity are enhanced by using 20–40 nm particle size and increasing silica concentration in the PNC.

In addition, this approach eliminates the need for elevated temperatures in the MOF’s development, since a uniform growth of ZIF-8 may be obtained at room temperature, as seen in the following example. For instance, Guo et al. [148] employed this approach to produce a PNC of MOFs at room temperature to remove Rhodamine B dye molecules from wastewater. Using this method, a coating of ZIF-8 was applied to the hollow PVDF fibre’s macroporous surface. All the components were submerged in a methanol/water solution for 12 h at room temperature. The preparation was then submerged in Zn(NO_3_)26H_2_O in an ethanol/water solution at 30 degrees Celsius for six hours. Thus, the creation of ZIF-8 membranes on the inner or outer side of hollow PVDF fibres may be regulated so long as the pre-coating of one or the other surface with the Zn precursor or ZIF-8 seeds is regulated.

#### 5.1.3. Physical Combining

**Figure 4:**
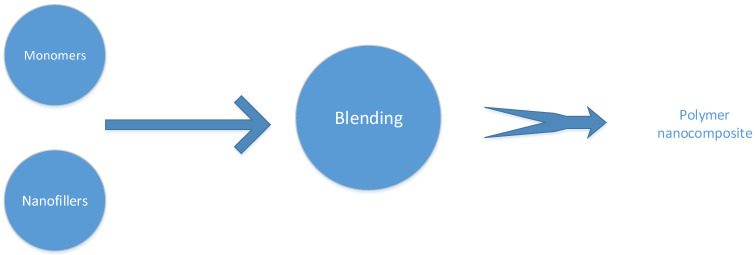
Steps to fabricate a PNC using the physical blending method.

Physical blending or casting is regarded as the easiest technique for producing hybrid polymer membranes. In this procedure, nanofillers and polymer matrix are combined directly (Figure 4). There are two primary ways to obtain the mixture: solution blending and melt blending. These two blending techniques are further examined independently.

##### Solution Blending

Solution blending or solution casting is a simple and efficient process for producing PNC membranes from any inorganic material. Moreover, the concentrations of chemicals in the combination may be readily regulated [149]. However, membranes may accumulate inorganic components [150]. In order to dissolve the inorganic material, the polymer must be soluble in the solvent. Stirring in three consecutive phases allows the two compounds to be mixed and distributed: (a) dissolving the polymer and then adding the nanofillers, (b) dissolving the nanofillers and then adding the polymer, and (c) dissolving both species to combine them. The polymer chains intercalate into inorganic structures by exchanging with solvent molecules. The membrane is then formed by evaporating the solvent by precipitation or under vacuum [151]. However, owing to health, financial, and environmental issues, this technique’s commercial viability is diminished by its high solvent use. Nevertheless, water-soluble polymers, such as poly(ethylene glycol) and polyvinyl alcohol, may be used with this approach in a water solution. Moreover, low or non-polar polymers may be employed to produce intercalated nanocomposites using this technique [129,131].

Panwar et al. [132] noted that by immersing the clay in the proper solvent, the solvent molecules permeate and extend the clay channels. However, they also noted that one of the main advantages of this procedure over melt mixing is the reduction in viscosity. This facilitates the movement of polymer molecules to the platelet surface. However, the solvent is also adsorbed on the clay’s surface, thus the polymer must better adsorb on the clay’s surface in order to displace the solvent. This last argument demonstrates that this procedure is superior for producing hybrid membranes from weak polar polymers.

Ragab et al. [152] removed micropollutants from water using a polytetrafluoroethylene (PTFE) double-layer membrane with ZIF-8. The PTFE membrane was dipped in various concentrations of ZIF-8 (20 wt.%) for the manufacture of hybrid membranes. In a comparison of modified and unmodified membranes, it was discovered that the changed membranes enhance the adsorption capacity of micropollutants by 40% while increasing their water permeability. This permeability is also highly intriguing since it would minimise the process’s energy usage. In addition, following three regeneration cycles using poly(ethylene glycol)-400, it was discovered that the membrane retained 95% of its original efficiency.

Low et al. [153] created a novel two-dimensional ZIF with a leaf-like architecture (ZIF-L). The influence of incorporating this ZIF-L onto polyethersulfone (PES) membranes on its ultrafiltration characteristics was investigated. The membrane modified with 0.5% ZIF-L yielded the greatest results, as its water flow was enhanced to 75% and its resistance to fouling with bovine serum albumin was almost doubled (BSA). These findings achieved with the modified membrane may be explained by the decrease in zeta potential, the rise in hydrophilicity, and the decrease in surface roughness, which made it more difficult for BSA to bind to the membrane surface.

##### Melt Blending

When it comes to synthesising PNCs, the melt blending method (also known as melt compounding, melt casting, or melt intercalation method) is by far the most popular, flexible, and favoured methodology. As an added bonus, the method is widely employed in industry since it is solvent-free and safe for the surrounding ecosystem. It is the most exciting method for PNC synthesis with industrial applications [124], and one of the best methods overall since it is compatible with cutting-edge industrial processing equipment such as injection moulding and extrusion. Since it does not need the selection of a solvent, it is also simpler than in situ polymerisation, sol-gel, or solution mixing [124,129,130,154] and is accessible for commercial polymers that are incompatible with these techniques. This process includes combining thermoplastic polymers and nanofillers using force. Various processing methods are available, including single- or double-screw extrusion, internal mixers, and manual mixing. The external pressures used during processing enhance the dispersion of nanofillers into the polymer matrix to be simpler and more uniform. Paul et al. [126] investigated the dispersion and intercalation of nanofillers in polymers and determined that shear forces due to extrusion melt processing and mixing conditions owing to screw speed, mixing duration, and temperature are both crucial characteristics. In addition, the type of the polymer, its molecular weight, and its polarity impact the interaction between phases, which is crucial for obtaining a homogenous filler dispersion [155]. The blended hybrid material undergoes annealing at a temperature greater than the Tg of the polymer, enabling the appropriate mixing of components for membrane fabrication. However, this feverish temperature might be a drawback since hot temperatures can occasionally breakdown polymers. Using solid-state NMR, Van der Hart et al. [130] studied different exfoliated nylon-6/clay membrane combinations and compared them to pure nylon-6. Using mixing and in situ polymerisation, the membranes were produced. The clays were first treated with a cationic modifier, which attaches ionically to the surface of the clay layers and causes them to expand. In terms of the spacing, crystallinity, and mobility of the non-crystalline nylon-6 segments, the findings obtained for modified hybrid membranes produced by both preparation procedures were comparable. Bhiwankar and Weiss [154] used tetra-octyl and tetra-decyl ammonium salts of sulfonated polystyrene (SPS) as ionic compatibilizers of polymer PS and pure Na-montmorillonite clay using this approach. After combining all the components, the compatibilizers exhibited excellent exfoliation and dispersion of the clay, most likely as a result of the separation created by the quaternary ammonium ion, which exchanges with the Na in the channels. In addition, it was noticed that exfoliation increased when the alkyl chain length of the counterion of the compatibilizer increased. In addition, the membranes containing the compatibilizer exhibited a larger storage modulus. Motamedi and Bagheri [156] examined the structure and characteristics of polymer composite membranes made with polypropylene (PP), nanoclay, and polyamide-6 (PA6) utilising this preparation process, creating PP/organoclay, PA6/organoclay, and PP/PA6 and PP/PA6/layered silicate combinations. The PA6/organoclay PNC had an exfoliated structure because the clay was contained inside the PA6 particles, resulting in an increase in viscosity. In contrast, the silicate layers in the PP/PA6 PNC were oriented near the PP/PA6 interface. In this sample, it was also noticed that the organic clay altered the form and size of the PA6 particles. Finally, the mechanical characteristics of the produced samples with two components were enhanced. In a separate work, Yoon et al. [155] examined the influence of polar comonomers in PS chains on melt intercalation in organosilicate galleries. The comonomer composition and two production techniques of styrene/organosilicate membranes were investigated. PS and three distinct styrenic copolymers containing methylvinyl oxazoline and acrylonitrile units were employed as polymers. At the same temperature, the two synthesis procedures were used with and without shearing. In every instance and very rapidly, the polymers intercalate flawlessly inside the organosilicate framework. The structural stability of polymer/organosilicate hybrids seems to rely on the interactions between both components. Because polymer chains do not diffuse effectively into organosilicate layers, the weak interactions between components resulted in an unstable hybrid. However, when polar comonomers were used, the resulting PNCs were stable because of the strong interaction between their constituents.

### 5.2. Electrospinning

Electrospinning is regarded as one of the most significant techniques for obtaining polymeric (or composite) nanofibers with diameters between the nanometric and micrometric scales [157]. One of its greatest benefits is its adaptability, which enables simple, inexpensive, and customizable membrane production with variable pore size and distribution, aspect ratio, elasticity, and stiffness, among others. Thin-film nanocomposites, mix-matrix hybrids, two- or three-layered composites, and metal–organic membranes with a broad range of components have all been produced using this approach [158]. Electrospun membranes have been used in a wide range of environmental applications, including MF [159], UF [160], NF [161], RO [162], oil/water separation [163], MD [32], and bio-separation [164].

The management of the structural integrity, stability, and functioning of electrospun nanofibrous membranes (ENMs) across a broad range of operating conditions, such as temperature, fluid pressure, and pH [158], is critical for their usage in a variety of applications. These features are integrally connected to the diverse polymeric or composite systems as well as the morphology/topology of the electrospun fibres. There are approximately 200 polymers that have been utilised effectively for the creation of fibres by electrospinning [165], providing a broad variety of compositions for membrane manufacturing. Moreover, electrospinning is compatible with a variety of inorganic fillers, such as nanoparticles (NPs) of various natures (SiO_2_, TiO_2_, ZnO, carbon nanotubes, etc.) [166,167]. The plethora of available combinations enables the enhancement and/or modification of membrane mechanical characteristics to meet a variety of operating situations and, in certain instances, to offer secondary capabilities such as photocatalytic activity [167]. Compared to other conventional fabrication techniques such as non-solvent-induced phase inversion (NIPS) and thermally induced phase inversion (TIPS), electrospun membranes have an interconnected open pore structure and an easily tenable thickness, giving them superior porosity and permeability [168].

#### 5.2.1. Instrumentation and Conceptual Framework

**Figure 5:**
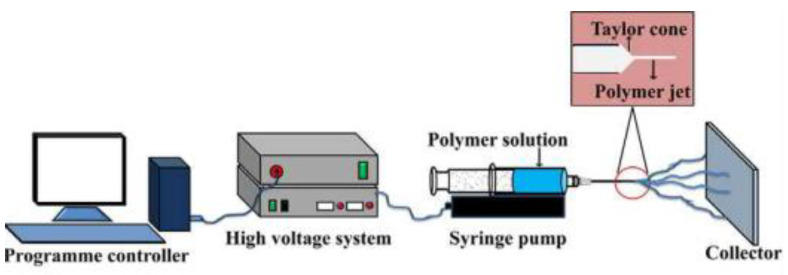
The simplest method of assembling an electrospinning machine [169]. Creative Commons BY-NC-SA.

A notable advantage of the electrospinning method is that the required equipment is quite simple (as shown in Figure 5), which makes the method easily accessible to almost any research centre. The following are the major constituents: (i) a static DC high-voltage power supply source between 1–30 kV, (ii) a syringe pump that allows the control of the fluid flux between 0.01–2 mL·h^−1^, (iii) syringes and needles, in most cases, hypodermic blunt tip type needles are used, with different lengths and inner diameters between 0.3–1 mm, and (iv) a metallic collector, in most instances, an aluminium one. It is possible to manufacture materials with nanofibers that are randomly oriented by setting the grounded collector so that it is perpendicular to the needle, or it is possible to produce materials with nanofibers that are oriented in the desired deposition direction by using a rotatory system.

Electrospinning is based on an electrohydrodynamic process in which a drop of polymer solution or melt is subjected to the electric potential difference caused by the electrical field in which it is immersed. This difference in potential is what makes electrospinning possible. Taylor’s cone is the name given to the shape that the drop takes on, which resembles a jet or a cone and is analogous to the point of a fountain pen. This cone goes through elongation and flapping processes because electrostatic tensile forces exerted on it are greater than the surface tension of the fluid it encounters. These pressures cause the polymer to be driven into a connected metal manifold, and it is in this manifold that the polymeric fibres that will eventually make up the final membrane are deposited and overlaid.

#### 5.2.2. Parameters of Control in an Electrospinning Process

**Figure 6:**
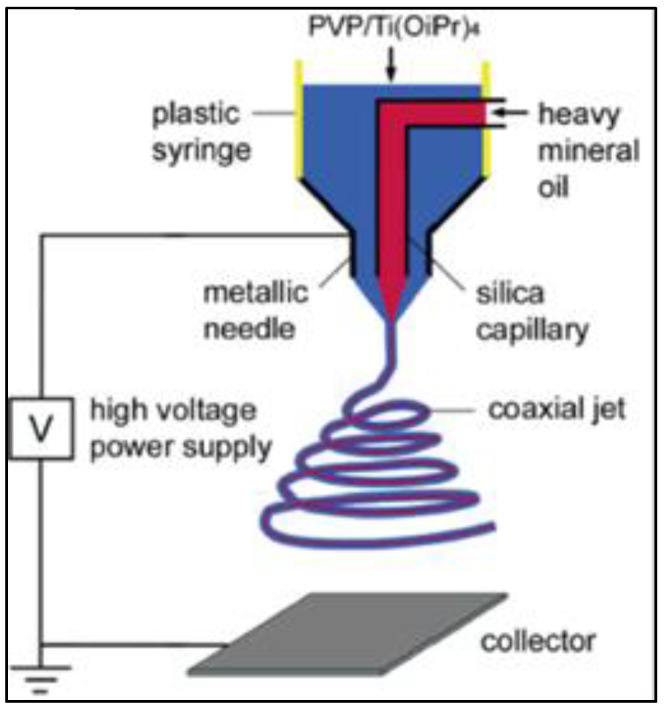
A coaxial electrospinning setup diagram. Li et al. [172].

Every step of the electrospinning process is determined by several factors, each of which has one of three unique qualities: (i) electrospinning characteristics, such as the needle inner diameter, the fluid flow velocity, the applied voltage, and the distance between the needle tip and the collector; (ii) the parameters of the fluid, such as its viscosity and conductivity, as well as, for solution electrospinning, the kind of solvent and the concentration of the polymer; (iii) environmental variables, including temperature and moisture [158]. The true difficulty of this technology is in the adjustment of these factors to produce fibres with a certain diameter and shape. Each of these factors has a distinct critical value for each distinct electrospinning system. Setting a parameter outside of this range will always result in fibres with undesirable morphologies, pearl-beaded formations, discontinuous fibres, or insufficient fluid solidification prior to deposition on the collector. The versatility of the electrospinning process is enabled by the simplicity of standard electrospinning equipment. Multi-needle electrospinning and coaxial electrospinning are examples [158]. Multi-needle electrospinning increases production by as much as 18 m^2^·h^−1^ [170] but adds additional control factors such as the number of needles, the distance between them, and their arrangement [171]. A coaxial spinneret, formed by two concentric needles, permits the extrusion of two distinct systems that, upon meeting at the needle’s tip, create a core-sheath structure (Figure 6) [172]. Coaxial electrospinning adds the parameter of miscibility between the two extruded fluids and makes it desirable for both fluids to have comparable dielectric characteristics [173] to provide comparable electrical pilings.

#### 5.2.3. Membrane Electrospinning of Composites

When it comes to generating membranes by electrospinning, these two methods are by far the most common ones to use. They both concentrate on composite nanofiber membranes. When creating ceramic-based composite fibres, it is common practise to add sol-gel precursors [174]. On the other hand, this is not always the case, and the approach that is utilised the most often for composite fibres with polymeric matrices is the integration of nanoscale components into polymeric solutions [175]. Over the course of the last several years, it has come to widespread public attention that viable composite nanofiber materials for use in water purification applications have been produced employing these two methods.

The many applications of nanocomposite membranes are outlined in Table 2, which may be found here. It is essential to recognise that the incorporation of the presented nanocomponents or any other nanocomponents will always have the ultimate effect of modifying the aspect ratio, the mechanical properties, the water flux [170], and/or the hydrophobicity of the material. Because of this, it is essential to optimise the proportion to be used to adjust these properties to their optimal values for each application. Chemical compatibility, contact angle, pore size, porosity, and surface roughness are some of the most important considerations to make when choosing the best appropriate composite system for a particular application [176].

### 5.3. The 3D Printing Innovation

Additive manufacturing or 3D printing technologies have emerged as extremely adaptable and promising approaches for a variety of applications. From aerospace [194,195,196], automotive [197,198], and construction [199] sectors to biomedicine [200,201] and the food sector [202], the range of fields in which this technology might be utilised has grown significantly. Even though it is currently relatively understudied, the number of membranes produced by 3D printing technology for desalination and water remediation rises annually.

In addition to the reduction in membrane fouling and the chemical stability of the membrane, one of the driving forces behind the search for more efficient and lucrative membranes is the necessity to remove new pollutants such as emerging contaminants. This requirement is one of the driving forces behind the search. A manufacturing process that can manage membrane structure has been adopted as an economical and solvent-free approach for the manufacture of new membranes thanks to the development and expansion of 3D printing technology. Therefore, three-dimensional printing, which is capable of producing complex structures despite their reduced size, has been identified as one of the most promising methods for the production of membranes in the years to come. However, this method still has significant disadvantages that hinder its widespread use. Table 3 highlights the benefits and drawbacks of the most used membrane production processes [203,204] to date.

**Figure 7:**
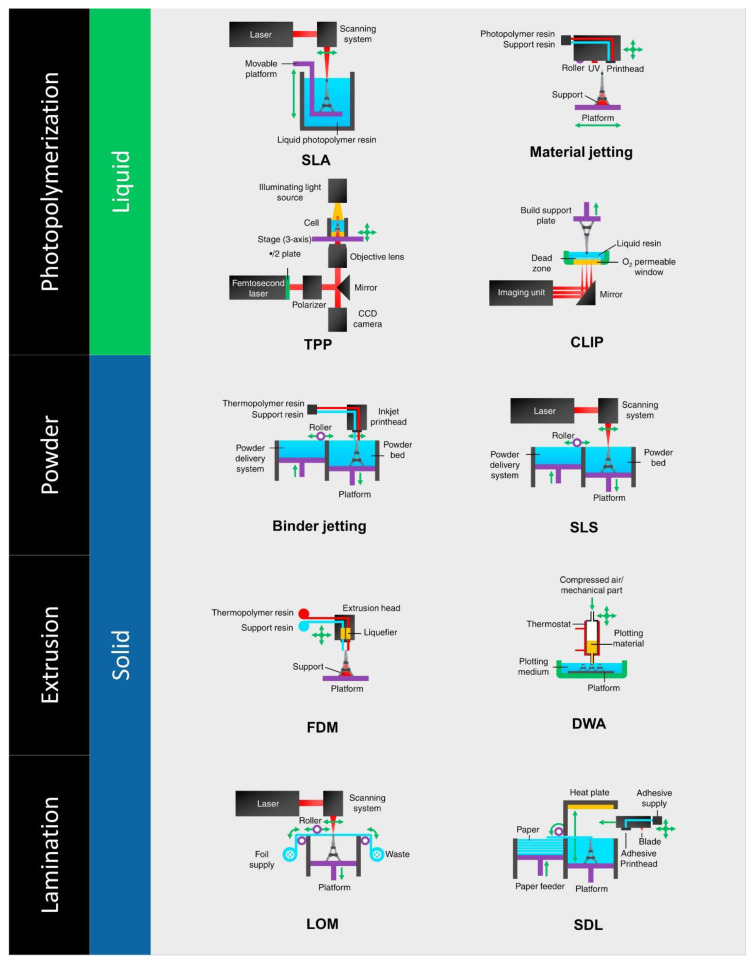
A summary of the major 3D printing organisations and technologies. Stereolithography (SLA), two-photon polymerisation (TPP), continuous liquid interface production (CLIP), fused deposition modelling (FDM), direct writing assembly (DWA) (DWA could be used in both liquid and solid), laminated object manufacturing (LOM), and selective deposition are all examples of techniques used in semiconductor manufacturing. Reproduced under the Creative Commons CC-BY licence from [206].

According to the material feed or deposition process, 3D printing technologies are often categorised into four categories (Figure 7): (1) powder, (2) material extrusion, (3) lamination, and (4) photo polymerisation. The most used 3D printing technology is fused deposition modelling (FDM), a filament extrusion process that typically prints thermoplastics (PC, ABS, PLA, etc.) with a resolution between 50 and 200 microns. In three-dimensional printing (3DP), selective laser sintering (SLS), selective laser melting (SLM), and liquid binding jetting are among the powder-processing-based techniques employed. In these techniques, the powder is applied to the construction platform and is used to strategically combine material layers. SLS resolution is close to 80 m, but 3DP resolution ranges from 100 to 250 m. Laminated object manufacturing (LOM) and selective deposition lamination (SDL) utilise cut and laminated polymer sheets as feedstock in lamination-based 3D printing. Lastly, photo-polymerisation-based 3D printing, also known as stereolithography (SLA), employs UV light to commence the polymerisation process, solidifying a polymer/resin layer that binds the following layers together. In this instance, the resolution is around 10 m. In addition to the popular 3D printing methods, additional printing technologies have been improved during the last several years. Among the revolutionary techniques, two-photon polymerisation (TPP) and continuous liquid interface production (CLIP) stand out because of their superior resolution and enhanced mechanical characteristics when compared to conventional techniques [205]. Moreover, these procedures are extensively used in several applications, but when it comes to membrane manufacturing, photo polymerisation has emerged as the most prevalent process [206].

Up to now, when contemplating 3D-printed materials for water clean-up, the prevailing technical limits, resolution beyond 10 m, have limited their usage to module spacer production rather than membrane synthesis [207]. Nonetheless, recent advancements in advanced manufacturing (AM) technologies have increased the viability of 3D printing for the production of membranes, and various instances of membranes based on this method have been published [208]. Koh and co-workers, for instance, produced an antifouling oil/water cellulose membrane by direct printing. The average pore size of the cellulose mesh produced was less than 240 m, and its separation efficiency was almost 95% [209].

This progress has also enhanced the creation of hybrid materials that can be printed using the many current techniques. Sangiorgi et al. [210] created hybrid polylactic acid (PLA) and titanium dioxide (TiO_2_) membranes using FDM technology.

## 6. Mechanism of Separation of PNC

The separation mechanism of conventional membranes and PNC is, in general, the same; however, the presence of nanocomponents on the surface and in the internal structure of the pores, or, in the case of dense membranes, the occlusion of the nanophase within the membrane can lead to minor changes in their descriptions. The impact of nanocomponents on separation based on the usage of PNC may be examined by examining the separation mechanisms or membrane performance. In general, a large number of papers [119,211,212,213,214] have concentrated on antifouling qualities and operational performance (i.e., the improvement of mechanical and thermal properties for their use in operations at high temperatures and high pressures). Therefore, the rejection coefficient and permeability of a porous polymeric membrane are the characteristics that characterise its use in the separation of analytes dissolved in water. Because of this, the description of the process contains the initial values of the rejection coefficient and permeability at a starting temperature that has already been established. Alterations in the temperature at which the process is carried out contribute to an increase in permeability because temperature has a direct correlation to permeability. Despite this correlation, an increase in temperature causes the polymer phase to expand, which in turn results in an increase in the effective pore size. If size exclusion is the mechanism of separation, then an increase in effective pore size will lead to a large fall in the rejection coefficient. This will be the case if the rejection coefficient decreases. In contrast, if a porous polymeric membrane is nanostructured, its thermal characteristics may be enhanced, and as a result, it may be conceivable for PNCs to increase their permeability in response to an increase in temperature without compromising their rejection coefficient values. Globally, the membrane performance is enhanced in PNCs compared to non-nanostructured membranes because of this example. However, the enhancement of attributes is contingent on the type and concentration of the nanomaterial, the properties of the polymeric phase, and the interaction between the nanostructured and polymeric phases.

**Figure 8:**
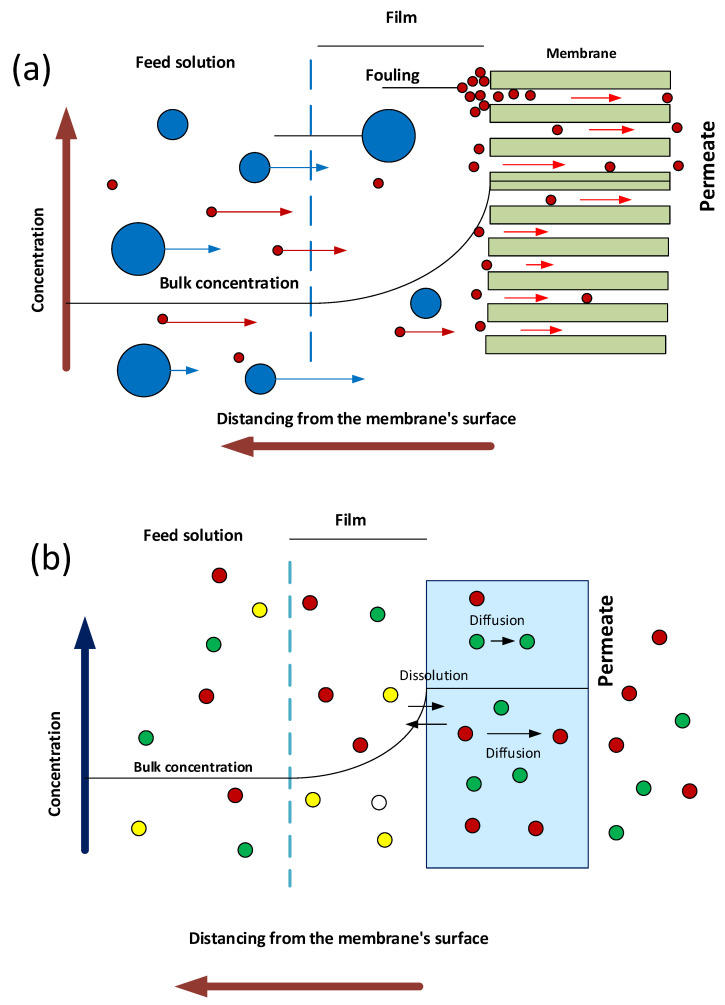
Separation by size-based exclusion (**a**) and dissolution–diffusion (**b**) methods.

The examination of separation processes is essential for a complete comprehension of PNCs. In general, two mechanisms can be identified for membrane separation processes: (i) separation by size-based exclusion and (ii) separation by dissolution–diffusion [120]. In the first case, particles that are larger than the pore in the membrane are retained, while those that are smaller are allowed to pass through. This process takes place in membranes, referred to as nano-, ultra-, and microfiltration (NF, UF, and MF, respectively), with water being the majority of the fluid phase. The input stream’s liquid phase might be homogeneous (e.g., aqueous dissolution for macromolecule removal) or heterogeneous (e.g., aqueous dispersion for the removal of suspended solids). In some settings (e.g., air filtration), it may be seen as a gaseous fluid containing suspended particulates [120,215]. During the second step, known as separation by dissolution–diffusion, the substance that penetrates the membrane is first dissolved inside the membrane (during the stage referred to as the dissolution stage), and then it diffuses across the membrane (i.e., diffusion stage). This mechanism is used to describe the separation process in dense membranes (for example, bulk, emulsion, and supported liquid membranes), but it can also be used to describe the separation by porous membranes of reverse osmosis (RO). This is due to the fact that the nanoconfinement of matter in the inner of very small pores increases the interaction between the permeant substance and membrane phase [120,215]. Figure 8 depicts the main processes of membrane separation techniques.

### 6.1. Impact on the Size Exclusion Mechanism

Understanding the effect of nanometric components on porous PNC separation requires looking at the process, rather than focusing just on the membrane itself. Because of this, the composition of the membrane is of no consequence to the procedure of separation if a system is envisioned in which the fluid and the species contained within it do not interact with the membrane, which may be an NF, MF, or UF membrane. In this scenario, the nature of the membrane is irrelevant. Regardless of whether the membrane is ceramic, polymeric, or nanocomposite, the separation will be controlled by the cut-off of the membrane, which is determined by the pore size and particle size distribution in the fluid. This means that ceramic, polymeric, and nanocomposite membranes all have the same effect on the separation. It is important to note that this hypothetical “zero interaction membrane” or “non-interacting membrane” should not be confused with the concept of “zero retention membrane”, which is used for the modelling of hybrid methods of membrane separation such as polymer-enhanced ultrafiltration, micellar ultrafiltration, and liquid membranes enhanced with liquid-phase polymer-based retention [216,217]. This is because the separation mechanism being analysed is size exclusion, as the process involves the addition of liquid membranes that are enhanced

**Figure 9:**
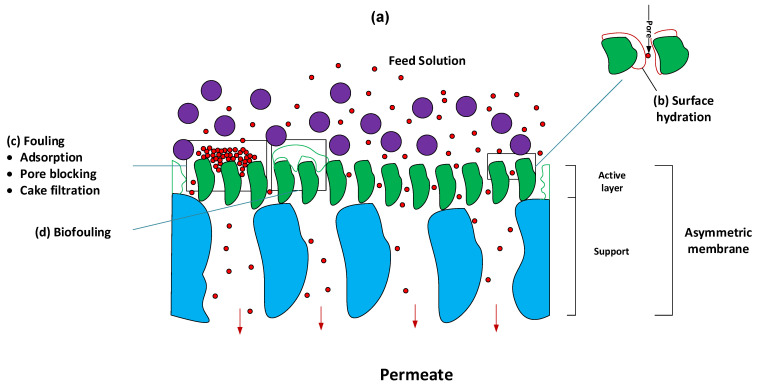
(**a**) Diagram showing numerous processes that occur during membrane separation through the size exclusion mechanism, (**b**) surface hydration (solvent = water), (**c**) fouling, and (**d**) biofouling.

On the other hand, when the membrane interacts with one or more fluid components, the nature of the membrane becomes a primary component if the interaction is significant. This is due to the fact that the nature of the membrane is associated not only with the type of interaction that occurs between the membrane and solutes, but also with the phenomena that are triggered as a result of these interactions. These may be both good and negative occurrences, with the major negative consequence being an increase in fouling and, as a result, a loss in operating capacity due to decreased permeability and selectivity. Positive benefits might include less fouling, increased permeability and disinfection, longer working durations, fewer secondary cleaning stages, a longer half-life time, and improved microbiological quality of retentate and permeate [113,114,115,116,117,118] (see Figure 9).

**Figure 10:**
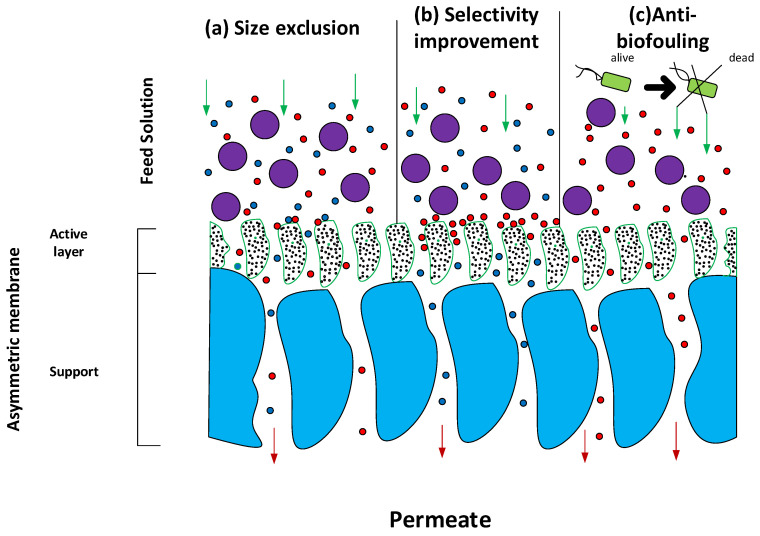
(**a**) A schematic of a nanocomposite asymmetric membrane that does not affect separation mechanisms; (**b**) an improvement in selectivity due to the interaction of a surface nanomaterial with a particular solute; (**c**) an improvement in functionality due to the presence of anti-biofouling properties.

When a PNC that is not interacting with anything else is investigated, however, various cases may be found, including the following: (i) If the nanomaterial that makes up the PNC only improves its mechanical and thermal characteristics, it is predicted that the membrane’s permeability will improve, but there will be no effect on the mechanism that controls separation, and as a result, the PNC will not be an active component when it comes to the separation process (see Figure 10a). (ii) If the nanomaterial that makes up the PNC improves the performance of the membrane and the separation process, then there is some kind of beneficial interaction with one or more solutes. For instance, PNCs that are based on silver nanoparticles (AgNPs) are an active element during the separation process because they reduce biofouling. One or more additional interactions at the level surface may take place in comparison to the equivalent non-nanostructured membrane. This is due to the fact that the surface of the nanocomposite that makes up the active layer of the membrane in these systems has AgNPs that are partly exposed and come into contact with the feed stream. In the case of AgNPs, the nanoparticle surface chemically links molecules that have thiol groups on their structures. As a result, even though small molecules such as cysteine, which have dimensions of 0.64 nm (length) and 0.37 nm (width) [218], cannot be removed by the size exclusion mechanism using NF, UF, or MF membranes, the formation of RSH-Ag0 bonds can produce the retention of cysteine molecules without causing changes to them. In addition, washing the membrane under certain pH conditions might facilitate the recovery of cysteine that is linked to the membrane [219,220] (see Figure 10b). In addition, the anti-biofouling characteristics of PNCs based on AgNPs have been comprehensively established in several studies throughout the scientific community. AgNPs, in general, have the potential to function as a reservoir for Ag^+^ ions, diffuse off the surface of the membrane, and enter the bacterium, where they interact with a variety of biomolecules, ultimately leading to the death of the microorganism [217] (see Figure 10c).

In these membranes, AgNPs are reduced and stabilised using an appropriate stabilising agent (e.g., citrate, glucamine, PVA, etc.) before being added to the polymer phase to generate the nanocomposite, which is then employed to make the membrane. In addition to the preferential retention of thiolate compounds and anti-biofouling capabilities, it is clear that the addition of AgNPs impacted other material properties, including dielectric properties. There have not been many studies that have focused on describing the dielectric response of PNC in terms of the chemical composition of the polymer phase and nanomaterial, despite the fact that this type of membrane has the potential to be used as a sensing surface for both monitoring a particular effluent and performing process self-control operations [221,222]. To produce anti-biofouling membranes or anchoring surfaces of thiolate compounds, AgNPs can only be added at the surface level. However, this type of membrane could be referred to as “nanostructured” and “active”, but not a PNC, because the active polymer phase is not a nanocomposite. It is important to note that AgNPs can only be added at the surface level.

### 6.2. Influence on the Dissolution–Diffusion Mechanism

Liquid membranes are not included here since the research presented below is restricted to polymeric membranes alone. The dissolution–diffusion mechanism may be broken down into two main stages: the first step is the dissolution stage, and the second stage is the diffusion stage. It is essential to keep in mind that the dissolving stage is determined by the phases that are in touch with one another; hence, it is inextricably linked to the constituent parts of the solute and the membrane. Interfacial contact is favourable and the transfer of analytes to the membrane phase is promoted if the feed phase has a high affinity for the membrane phase. This step is comparable to size exclusion because of the fact that size is used when it is specified as a criterion for affinity. Particles that are larger than the membrane cut-off are unable to enter the membrane phase, therefore their affinity for the membrane phase is low. On the other hand, particles that are smaller than the membrane cut-off have a high affinity for the membrane phase since they can enter it. In a similar manner, the dissolution of analytes in the membrane phase serves as an exclusion criterion. As a result, solutes that have a weak affinity for the membrane phase are not allowed to pass through it, while analytes that have a strong affinity are permitted to do so. This affinity is connected to the structural similarity of the membrane-forming polymer and the solute, or, to put it in molecular terms, to the collection of intermolecular interactions that take place between the membrane material, the analyte, and the transporting phase [120,223,224]. Because of this, it is challenging to understand how the nanomaterial will affect the very first step. However, further investigation reveals several fascinating components that contribute to a better understanding of the mechanism behind the process.

To elaborate on the idea presented previously, take into account the simplest system that could possibly exist, which would be equivalent to a feed that did not contain any solutes. In this scenario, for the dissolution stage, a single interaction should be analysed for both non-nanostructured membranes and PNCs, and that interaction is the solvent–membrane interaction. The fluid phase in the feed is referred to as the “solvent.” In the case of gases, the analysis is limited to the examination of a single gas that is allowed to pass through the membrane. If the molecular diameter of the gas molecules was smaller than the cut-off for the membrane, then the gas molecules would have a strong affinity for the membrane in the case of porous membranes such as RO membranes. In very certain situations, such as when the gas molecules are enormous in relation to the cut-off point of the membrane, the affinity may be significantly diminished. However, the affinity in dense membranes, which are crosslinked polymers that are embedded in a liquid, is governed by the solubility of the gas in the liquid; hence, the structure of the polymeric material is analogous to that of a hydrogel when water is the solvent. However, the solubility of the gas will be determined by operational factors such as pressure and concentration (chemical potential), which are the fundamental characteristics that have an impact on the gas’s Henry constant. This will be the case because pressure and concentration are the fundamental characteristics that impact the gas’s Henry constant. [120,223,224].

In the case of thick membranes, however, if the feeding stream is a liquid, three scenarios are possible: (i) the feeding phase has a high affinity for the liquid contained in the membrane, (ii) the feeding phase and the liquid in the inner of the membrane are the same, and (iii) the feeding phase has no affinity for the liquid in the membrane phase. The solvent will be able to infiltrate the membrane in the first situation, and the membrane’s stability will be governed by the mechanical strength of the polymer network. The nano-structuring of the membrane becomes critical at this time. In the second situation, nothing changes. As a result, in order to deepen the analysis, at least one solute must be included in the simplification performed. As a result, if the solute is physically like the medium and the medium is structurally similar to the membrane, the solute will have a high affinity in the membrane phase. However, in the scenario when the solute carries a net charge, the membrane surface features may be exploited to offer better selectivity to this initial step. In this way, if the membrane has charged, even if two solutes have a high affinity with the medium and the medium contained in the inner of the membrane has a high affinity with the membrane, it is possible to increase the rejection of one of the solutes with respect to the other by selecting the appropriate polymer structure via the use of an anionic barrier to prevent negatively charged particles from passing through. In the third instance, when the liquids are not structurally connected, the solute affinity is determined by the partition or distribution coefficient between the phases [120,223,224].

In the case of porous membranes, more specifically RO membranes, the solvent will penetrate the structure of the membrane, which will lead to two outcomes: the polymer phase has a high affinity with the membrane, and as a result, it is possible that strong solvation interactions will occur, which will promote the decrease in the permeability, which will result in a decrease of the effective pore radius; however, it is also very likely that the membrane will exhibit poor mechanical performance under these conditions. In circumstances such as these, the nanocomponent of the PNC contributes to the attenuation of the effect [101,102,103,104,105,106]. Because there is no affinity between the solvent and the membrane, solvation does not take place, the membrane material’s mechanical properties remain unchanged, and solute dissolution in the membrane phase can be easily understood by using the solute–membrane affinity in terms of relative size in relation to the membrane cut-off.

## 7. Nanocomposite Membrane Stability

**Figure 11:**
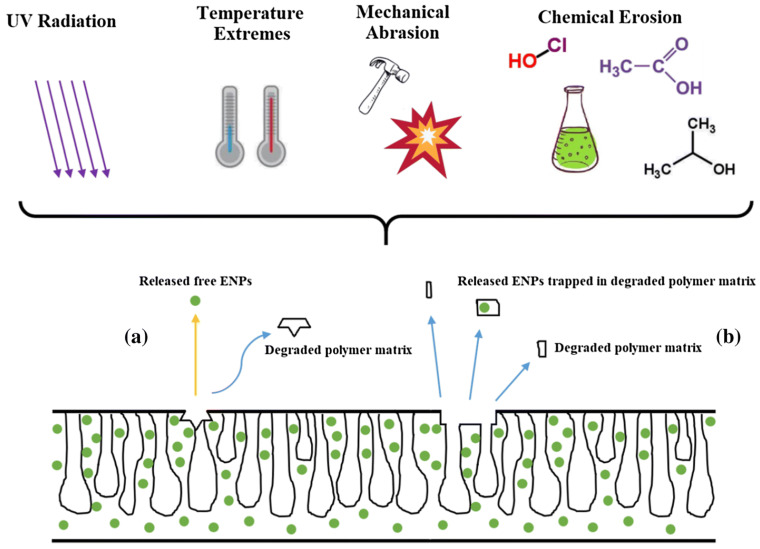
UV radiation, temperature extremes, mechanical abrasion, and chemical erosion as the four modes of polymer matrix deterioration. There are two ways that engineered nanoparticles (ENP) might release: either as (**a**) free particles when the polymer matrix degrades or as (**b**) particles combined with the destroyed polymer matrix.

Although nanocomposite membranes have shown several surprising features, their long-term durability remains questionable [225]. After a very short filtering time, a nanocomposite membrane was shown to lose 10% of its silver nanoparticles, resulting in a considerable decrease in antifouling and antibacterial activity owing to Ag^+^ leakage from the membrane surface [226]. Direct leaching, nanoparticle release, and degradation of the polymer matrix are the three processes that account for most nanoparticle leakage from nanocomposite membranes. Because the matrix fixes the nanoparticles and acts as a barrier to shield them from the outside environment, the chemistry of the polymer matrix is an extremely important factor that plays a significant role in determining the degree to which nanoparticles in nanocomposite membranes are stable. [227] Research has shown that carbon nanotubes may be connected to polyamide membranes in a way that makes them very stable. There are seldom any reports of carbon nanotube leaching from nanocomposite membranes. If the polymeric membrane does not undergo any structural or chemical changes, then it is reasonable to believe that carbon nanotube leaching is a very uncommon occurrence. Figure 11 demonstrates that UV degradation, temperature extremes, mechanical stresses, and chemical erosion are the primary mechanisms that lead to membrane deterioration [227]. When compared to the raw membrane, the polyamide nanocomposite membrane that included carbon nanotubes exhibited significantly improved resistance to UV radiation and temperature [228]. In addition, carbon nanotubes have the ability to operate as nucleating agents, which may change the lamellar orientation, dispersion, and viscosity of polyamide membranes, ultimately leading to an improvement in the membranes’ thermal and mechanical properties [228]. Therefore, the most likely source of nanoparticle release from damaged membranes is chemical erosion brought on by chlorine disinfection [227] (see Figure 11).

In order to maintain the functioning of nanocomposite membranes, a number of different methods for immobilising nanoparticles inside the membrane have been researched. One way is the synthesis of nanoparticles in situ, or “in situ”, inside the membrane matrix. It has been observed that polydopamine deposition and in situ reductions of silver ammonia aqueous solution (Ag(NH_3_)_2_O) are effective methods for immobilising silver nanoparticles in the polysulfone membrane [225]. It is possible that having smaller particle sizes may limit membrane nanoparticle leaching [229,230]. It was found that micro-sized silver had a leaching rate that was three times higher than that of silver nanowires and silver nanoparticles. During the process of water filtration, a greater particle mass of micron-sized silver particles caused a greater amount of silver to leach from the composite membrane. However, the antifouling properties of silver nanoparticles are partly due to the oxidation and release of Ag^+^, and as the size of silver nanoparticles decreases, more Ag^+^ ions will be released. This is because the smaller the silver nanoparticles are, the more Ag^+^ ions will be released. When an optimal silver size distribution is sought, it is necessary to strike a balance between the release of Ag^+^ and the detachment of silver nanoparticles from nanocomposite membranes. In addition, the interaction between chemical additives and nanoparticles that are contained inside the membrane has a major impact on the leaching of nanoparticles. It has been suggested that the incorporation of Pluronic F127 into a nanocomposite membrane that already contains titanium oxide nanoparticles may effectively limit nanoparticle leaching [231]. The interaction between additives and nanoparticles may assist reduce leaching by reducing the amount of nanoparticle aggregation that occurs inside the membrane. Polyacrylic acid was added to a poly(vinylidene fluoride) membrane that included Fe/Pd bimetallic nanoparticles [232], and the membrane became more stable. The presence of the linking molecule allowed for a more even distribution of the bimetallic nanoparticles across the membrane holes of varying depths [233]. In addition to the ways that have been mentioned above, an additional method that was studied as a viable strategy for minimising leaching was the pairing of a relatively unstable nanoparticle with a relatively stable nanoparticle. For instance, zeolites were covalently bonded to the surface of a polyamide membrane to act as anchors for silver nanoparticles [234]. After use, the membrane could be regenerated by simply physisorbing the silver nanoparticles, and the regenerated membrane showed antifouling performance that was comparable to that of the original membrane.

The chemical stability of nanoparticles influences the stability and performance of nanocomposite membranes in addition to their physical stability. Graphene oxide, in particular, may function as a terminal electron acceptor and be reduced to graphite by Shewanella [235], a genus of heterotrophic and metal-reducing anaerobes found in a range of settings. The decrease may also occur rapidly (24 h) in aerobic conditions [236]. It has also been reported that E. coli may reduce graphene oxide by glycolysis in 48 h under anaerobic conditions. Although graphene oxide is known to be antibacterial, the sheets may provide a biocompatible surface for E. coli to adhere to and thrive on [237].

To produce high-performance nanocomposite membranes, the uniform dispersion of nanoparticles in the polymeric matrix is a further significant difficulty. Due to high interactions between nanoparticles and weak polymer–nanoparticle interfacial contacts, nanoparticles tend to agglomerate. Poor nanoparticle dispersion and aggregation may result in modest improvements in nanocomposite membrane performance [238].

## 8. Membrane Hydrophobicity/Hydrophilicity

Because of the differences in the material composition and surface chemistry of the membrane, its affinity for water, and therefore, its wettability, might change. One of the surface characteristics of a membrane that might vary depending on the material is its wettability. The wettability of the material may be evaluated using the material’s surface tension to determine how well the material will behave when it comes into contact with liquid. It is possible to estimate the surface tension of a material by taking a measurement of the contact angle that exists between the surface of a membrane and a drop of liquid that is placed on the membrane. The potential of all substances to lower their surface area in reaction to an imbalance in molecular forces is what causes surface tension. This is because of the attraction between molecules. The surface tension of different liquids and solids may be measured, which can show differences between different systems. Hydrophilicity is a property that may be seen in materials that have a strong affinity for water. High surface tension levels are characteristic of materials that have a natural tendency to be hydrophilic. The chemistry of the material’s surface makes it possible to wet the substance by forming a film or coating of water on the surface of the material. On a hydrophobic surface, the water droplet will gather together to form a bead [239]. Materials that are hydrophobic have a low or non-existent propensity to absorb water. Hydrophobic materials have low surface tension values, and their surface chemistry does not include the active groups required for the creation of “hydrogen bonds” with water molecules. This makes hydrophobic materials less likely to interact with water. The wettability of a membrane is the most critical factor to consider when selecting the ideal membrane to use in applications involving water filtration. When it comes to the purification of fluids that include water, hydrophilic membranes are often used [239]. It is possible that the hydrophilic quality of nanocomposite membranes will change if nanofillers are included into polymer materials. To increase the membrane’s hydrophilicity, hydrophilic nanoparticles were incorporated into the membrane. When creating hydrophilic nanocomposite membranes, nanoparticles such as TiO_2_, graphene oxide, carbon nanotubes, silica, and cellulose nanofibers are among the ones that are used [240,241,242,243,244,245,246,247,248,249,250,251].

## 9. Aspects of Nanocomposite Membranes in Water Purification That Present Difficulties

The use of nanocomposite membranes in water filtration has followed a lengthy road of development. In reality, an abundance of bench-scale research investigations has been undertaken on the subject. However, it may seem contradictory that only a small number of water filtration systems based on nanocomposite membranes have been implemented in practise.

In terms of hybrid material efficiency, the gap between laboratory- and industrial-scale production poses the greatest challenge. Academic research tends to idealise, overvalue, and overestimate the performance of nanocomposite membranes, as well as to make a number of lofty promises regarding their industrial scaling up that would fall by the wayside if their potential limitations, such as technical practicability and feasibility, are not considered. Consequently, it is of paramount importance to avoid the scientific and industrial communities from being deceived by the transition to an industrial large scale, and this is accomplished by recognising the most common obstacles and issues that may be encountered.

In this section, we focus on the most significant obstacles facing the future uses of nanocomposite membranes in the water purification industry.

### 9.1. Scalability

Membrane fouling is a key barrier to the commercialisation of membrane technologies. Fouling is an undesired process of membrane film formation caused by the deposition of colloidal particles, organic materials, and microorganisms such as viruses, bacteria, fungi, and extracellular biopolymers (lipids, proteins, lipoproteins, glycoproteins, and polysaccharides). Biofouling by microorganisms is the most severe sort of fouling because these materials first cling to the surface, then grow on the membrane’s surface, and then multiply and proliferate in the presence of nutrients [252,253]. Extracellular chemicals are released from them, assisting in their growth. Membrane fouling reduces membrane lifespan and performance while increasing energy usage. To circumvent this issue, membrane materials that inhibit foulant adsorption to membrane surfaces are used [254]. Backwashing, high-pressure forward washing, and chemical cleaning are used to combat fouling. Fouling may also be minimised by using various membrane surface modification approaches. Modifications are made to change the wettability, surface charge, and surface roughness of a membrane. The hydrophilicity of a membrane may be boosted by combining polymer and nanomaterials to generate a polymeric nanocomposite membrane with higher salt rejection, water permeability, and permeate flow [255,256]. Nonetheless, despite recent advances in membrane technology’s success, some difficulties remain unsolved. The flux-selectivity “tradeoff” connection persists, and poor permeation rate remains a significant challenge impeding commercial use. This problem will be overcome once we understand the function of NPs and their interfacial interactions with polymer matrices.

Almost all research investigations using nanocomposite membranes for water treatment are currently undertaken on batch size. The process by which research and development moves from the laboratory to practical use in industry is referred to as “scaling up.” This is an important stage in the research and development process (R&D). Scaling up is not only a numeric concept that involves increasing the batch size; rather, it is a problem concerning the viability of the process (whether it is scalable or not), its productivity, and its effectiveness, all of which need to be explored in great detail. These batch tests serve a crucial role in giving a preliminary description and understanding of the depollution process using nanocomposite membranes but do not completely represent the actual yields at higher scales. The gradual transition from lab size to big practise is a result of the complexity of large system operations.

The design and manufacture of nanocomposite membranes for the removal of specific pollutants from simulated polluted water at the lab scale involve minimal amounts of materials and chemicals, as well as lower energy consumption. This is not the case at a bigger scale, when several factors might interact and need extra processing in addition to requiring substantial process adjustments. In addition, other disciplines, such as engineering, economics, and materials science, may be integrated to sustain industrial applications’ efficacy and satisfy rising needs and expectations.

Several approaches may be undertaken to improve the profitability of nanocomposite membranes’ performance and facilitate their sustainable use in the water depollution industry:

- Methods of synthesis examined to obtain a better result.
- Engineering suitability studies and economic analyses, including the structuring of scalable models.
- Effective quality control and applicable analytic techniques across all process phases to ensure quality.
- Comparison of the purity profiles of the initial and final materials.
- Production of large, homogenous amounts of well-characterised nanocomposite membranes for key prototype phase testing, and hence industrial evaluation.
- Excellent record of technology transfer techniques.

### 9.2. Stability

Nanotechnology is still in its infancy, and scientists are attempting to create novel techniques for enhancing the dispersion of nanoparticles. It is impossible to foresee the working principle of NPs since their behaviour and influence on society are determined by their interactions with their surroundings. However, we have a wealth of information on the use of nanocomposites from which to determine their environmental effect. Researchers are focusing on nanoscale methods and their applicability in the real world, as well as improving the characterisation of NPs. Therefore, there is a significant knowledge vacuum about the influence of NPs on the atmosphere. This is very necessary in order to comprehend the results of the experiment as well as their behaviour. Since 2007, the Organization for Economic Co-operation and Development (OECD) has been collecting data on the impact that NPs have had on the surrounding environment. The OECD has developed guidelines for the production and testing of products. The agencies in charge of regulation are still conducting risk assessments for composites that incorporate nanoparticles. It is of utmost importance to create a connection between the laboratory and the outside world.

The sorption and catalytic efficiency of nanocomposite membranes are insufficient to fulfil the anticipated demand on a bigger scale. The exceptional structural stability and high fatigue resistance of nanocomposite membranes, particularly in hard water conditions, is the primary factor driving the progression of water purification technology as well as the perception of the quality of purified water.

Since nanocomposite membranes are composed of two or more components, their stability reflects first and foremost the long-term assembly of all their components and their active components without any leaching in the reaction media. The second goal is to maintain the same level of dependability and efficacy throughout time. Recognising and comprehending the variables that might contribute to the instability of nanocomposite membranes is crucial for preventing their emergence and bringing stability to the materials even after several reuses.

According to the majority of the available literature, performance and stability evaluations of nanocomposite membranes are often conducted in simulated circumstances that closely resemble real-world conditions, but in short-term experimental experiments. The ins and outs of the long-term influence on the decontamination mechanism of nanocomposite membranes and their stability must be thoroughly investigated. All of this is meant to serve as the foundation for future implementations on a bigger scale.

Typically, the following methods are used to test the stability of nanocomposite membranes:

- Using analytical technologies such as atomic absorption spectroscopy (SAA) and inductively coupled plasma emission (ICP) [257] to investigate the leaching behaviour of nanocomposite membranes into the aqueous medium.
- Utilising X-ray photoelectron spectroscopy, or XPS, to investigate the binding energies, chemical composition, and atomic percentages of nanocomposite membrane components both before and during the treatment of wastewater with nanocomposite membranes. [258,259].
- Determining the magnetisation at saturation for hybrid magnetic materials [259,260].
- The excellent stability of nanocomposite membranes creates a double-paned window of enticing possibilities for their industrial uses. Reusability and regeneration for extended cycles are ideal examples.

### 9.3. Recyclability and Renewal

The reusability and regeneration capabilities of nanocomposite membranes are essential not only for their economic viability but also for their simple use on a broad scale. It is recommended that careful consideration should be given to these two criteria when designing nanocomposite membranes in order to obtain the most out of the significant amount of time and money that is required to construct these materials. The two criteria together constitute a substantial source of value addition to nanocomposite membranes.

When we talk about the reusability or recycling of materials, we are referring to their ability to be reused on several occasions up to the point when they become saturated and overloaded with water pollutants, which causes a decline in the efficiency with which they remove impurities. One solution instantly comes to mind: restoring the material’s key decontamination properties via its regeneration. This latter factor facilitates the reuse of nanocomposite membranes in the future, which serves as the foundation for possible cost-reduction prospects.

The recovery of adsorbate contaminants is essential, particularly in the case of dangerous heavy metals that must be desorbed and concentrated for continued industrial use rather than seeping into the environment. This is because the alternative would be for the metals to leak into the environment, which would have a negative impact on the environment and the economy. In order to analyse the reusability of materials at the lab scale, nanocomposite membranes are subjected to several cycles of repeated use under similar circumstances to obtain a clear understanding of the shelf life and maximum performance of nanocomposite membranes. In contrast, regeneration is assured by a variety of procedures that revitalise wasted, exhausted nanocomposite membranes in order to greatly reduce the need for virgin materials in favour of regenerated materials. Depending on the kind and formulation of the hybrid material, regeneration might include ultrasonic [261], thermal [262], electrochemical [263], chemical [264] treatments, etc.

Lab-scale testing of the hybrid material’s reusability and regeneration potential is a necessary precondition. However, the results produced from these trials are of limited use and may not always represent what may occur on a broader scale. The explanation for this is that most, if not all, laboratory-scale studies are conducted in batches. The move from static mode (lab-scale) to dynamic continuous operations (bigger scale) is often accompanied by a number of risk factors, including the influence of the flow rate and the harsh operating conditions of the larger scale, which may promote the deterioration of nanocomposite membranes. Consequently, their shelf life is drastically shortened compared to what was anticipated while in a static state.

Considering the aforementioned factors and in the interest of practicality, nanocomposite membranes should be able to withstand the most demanding environments and applications. Therefore, transitioning from a static, closed, and controlled system to a dynamic, open, and uncontrollable huge system should take into account more essential factors.

### 9.4. Interruptions

Pathogenic organisms, organic and inorganic pollutants, metallic elements, oils, radioactive elements, and so on, are among the many co-existing pollutants in contaminated water, none of which are regarded innocuous to human health [265]. In light of this, the performance of nanocomposite membranes may be assessed more accurately if they have been shown to remove many pollutants simultaneously.

Most experiments conducted in this vein with nanocomposite membranes were on the removal of water contaminants extracted from different solutions. However, few studies illuminate the significance of evaluating the influence of coexisting ions on pollutant removal [266]. It is not uncommon for coexisting inorganic ions to prevail at large concentrations in wastewater. According to recent scientific investigations, these ions have a considerable impact on the systems responsible for removing contaminants; they may speed up, slow down, or even halt the decontamination process.

Predictably, and according to the research, the presence of coexisting ions has a substantially detrimental effect on the removal of contaminants [264]. This is mostly due to the fact that competing interactions between the target pollutants and coexisting ions may occur at the binding sites of nanocomposite membranes, resulting in a decrease in affinity and decontamination efficacy.

As was mentioned before, polluted waterways such as wastewater (also known as sewage) contain a wide variety of different pollutants. It is essential to simulate wastewater at the lab scale in order to allow for a more accurate evaluation of the prepared nanocomposite membranes and to account for possible interferences within polluted water. This is carried out so as not to misrepresent the conditions of the contaminated water, but rather to fully describe them in order to infer the behaviour of the hybrid material. In order to accomplish this, it is necessary to simulate wastewater at the lab scale. After that, is the second step, which consists of testing the nanocomposite membranes on real samples of wastewater in order to carry out an in-depth investigation of their actual ability to clean water. In this manner, any and all potential interferences are already taken into consideration at the lab scale, in addition to the functioning of these materials; as a result, it is now possible to draw conclusions based on robust research and facts that can be relied upon.

### 9.5. Cost Efficiency

When evaluating nanocomposite membranes, both their ability to remove pollutants and their prices must be considered. The cost-effectiveness analysis is an essential component of environmental projects to maximise environmental benefits at the lowest cost, thus assuring an economically feasible option for large-scale water treatment.

The cost calculation of nanocomposite membranes for water treatment is seldom or never adequately specified in the evaluated literature. Estimating the cost of an elaborated material for a particular application may rely on a variety of parameters, such as the availability and pre-treatment of precursors, elaboration techniques and processing, the recyclability of the material, energy consumption, etc.

In the case of nanocomposite membranes based on bio-resources, such as biopolymers, their beginning components are readily accessible, natural, and affordable, allowing them to be used on a greater scale [267,268]. However, greater emphasis must be placed on the effective management and utilisation of natural resources in order to profit from the environment while maintaining its resources.

One of the key challenges that nanotechnology applications face when it comes to the purification of water is the use of nanocomposite membranes, which are composed of nanoparticles and nanomaterials. A major obstacle in using these membranes is the availability of copious quantities of these nanomaterials at acceptable rates. Consequently, it would be advantageous for the deployment of these materials as effective and enticing alternatives in the current traditional wastewater sector to locate suppliers who can meet the demands while giving cheaper pricing or at least within fair boundaries.

It is anticipated that the worldwide market for membranes would increase by 8.5% annually, reaching USD 26.3 billion in 2019. The development of improved membrane technology in combination with the use of nanomaterials makes it possible to create a greener, more sustainable future with less trash being produced all around the globe. PNCs that are of a higher quality and better techniques of separation may find use in the food, chemical, and pharmaceutical industries. The PNC-based separations’ excellent selectivity, low energy consumption, good cost-to-performance ratio, and tiny modular construction have contributed to their rise in popularity [269].

It is anticipated that membrane filtration will become the predominant method of water purification. Regardless of the enormous advantages, researchers must also account for the significant operating costs of the filtration plant, the environmental and resource impacts associated with nanoparticle functionalisation, and membrane maintenance [270,271]. Even though these difficulties may be resolved by enhancing membrane design, service, performance, and maintenance, there is an ongoing need to enhance and strengthen the sustainability of this technology [272].

It is vital to comprehend the rapid expansion of nanotechnology research and commercialisation in order to develop effective purification technologies. The commercialisation of nanotechnology in membranes often faces the following obstacles:

(i) Time delay: The commercialisation of nanocomposite-based membrane technology must not exceed three to five years [273].
(ii) Funding for research prototype development: There is a significant gap between obtaining funding for commercialisation and prototyping and a favourable research outcome. Compared to research costs, commercialisation expenses are high. Scientists do not focus on the actual application of their research, but businesses want a return on their investments.
(iii) A lack of necessary equipment: Research that is based on nanotechnology is very costly and requires very expensive fabrication equipment. The inability to promote the goods is hampered by a lack of equipment.
(iv) The lack of an assessment standard: The lack of performance evaluation criteria is a significant obstacle in nanoparticle preparation.
(v) Lack of financing: The commercialisation of nanoparticle-based membranes requires substantial expenditures that small- and medium-sized businesses cannot undertake.
(vi) Lack of qualified experts: Sufficiently qualified scientists, researchers, engineers, and technicians are desperately needed in this discipline.
(vii) Support from the general public: The general public is looking forward to novel scientific ideas such as nanotechnology. Therefore, companies who are interested in investing in this sector to deliver high-efficiency output obtain a stronger brand image, but sole proprietorships and small firms that now control a major share of membrane technology do not [274,275,276,277].

### 9.6. Persistence and Toxicity

Biodegradable, non-toxic, biocompatible, eco-friendly, ecological, etc., are the words most often used in the literature to describe nanocomposite membranes for water treatment [257,264,278]. To bridge the gap between these claims and reality, it is necessary to measure the sustainability and “non-toxicity” of these materials prior to their widespread use. However, there is a paucity of research on this topic.

Why are nanoparticles so dangerous? The solution lies in their modest size in comparison to lung alveoli (200 µm), blood capillaries (5 µm), red blood cells (2–6 µm), mitochondria (500 nm), and fenestrated capillary holes (up to 180 nm) [279,280]. Nanoparticles smaller than any of the aforementioned systems may be ingested by inhalation, enter the circulatory system, travel to organs, enter cells and even organelles, and influence fundamental cellular processes such as metabolism and cell death, hence causing illness [279,281]. Some nanoparticles cause oxidative stress and inflammation, denaturation of proteins, and damage to cell organelles and DNA [282,283,284]. Noting that compounds that are not harmful in bulk form may become toxic as nanoparticles is essential. As we have learned more about the intrinsic toxicity of most nanoparticulate materials in recent years, occupational exposure of employees to airborne particulates has become a more relevant area of study. According to studies [285,286,287,288], occupational exposure to nanoparticles may occur at all phases of manufacture, research, wear and tear, recycling, and disposal of nanoparticles and materials containing nanoparticles.

Few papers addressed the biodegradability or sustainability metrics of previously manufactured nanocomposite membranes by statistically and qualitatively investigating their possible environmental effects [278] or by evaluating their biodegradability [129]. In the case of adsorption, wasted nanocomposite membranes represent secondary waste if they are not regenerable. Indeed, even on a small scale, adsorbents containing harmful pollutants such as heavy metals are considered to pose significant risks to human health and the environment, and this risk would be amplified if the size were increased.

Numerous researchers are now eager to use nanocomposite membranes for the filtration of contaminated water due to these qualities. TiO_2_ is one of the catalytic nanoparticles often employed. Many environmental pollutants, such as PCBs (polychlorinated biphenyls) [289,290], halogenated aliphatics [291,292,293], aze dyes [294,295], halogenated herbicides [296], and organochlorine pesticides [297,298], are often degraded using catalysts or redox reagents. The challenge with this catalytic breakdown, however, is that it may lead to poisoning or the production of by-products that have the potential to infiltrate into purified water, which then results in the presence of dangerous compounds in the water. After embedding nanoparticles (NPs) of silver, zinc oxide (ZnO), and titanium dioxide (TiO_2_) in a membrane and evaluating them as an antibacterial material for water purification [299,300], it was found that toxic by-products may be created if a nanocomposite membrane leaks or interacts with water [300].

It is important to take into consideration the possible risks to one’s health that are linked with the absorption of NPs in drinking water in the event that NPs leak into purified water during the process of purification. The phenomenon of nanoparticle leaching is ignored in a significant number of research studies [301,302]. However, this is an indirect estimate suggesting the likely presence of NPs in water [303,304]. A small number of studies have assessed the total metals in treated water after filtration utilising contemporary nanoparticle treatment technologies. For instance, measurable quantities of silver particles were present in water that had been filtered using silver nanoparticles embedded into paper filters; however, the concentrations of silver NPs in the water were significantly lower than what is recommended by the World Health Organization (WHO) and the United States Environmental Protection Agency (EPA) [303]. Even after 15 h, ceramic filters that had been coated with nanoparticles of silver continued to leach silver into the drinking water [304]. Silver levels in early water samples taken during the first two hours of filtering exceeded 100 ppb [304]. These scientists did not determine if the silver ions or nanoparticles that drained from the passages were present. Nonetheless, the results indicate that the purifying technologies may not be beneficial. Even though these filters were effective at eliminating microbiological pollutants, it is vital to assess the hazards posed by the presence of low-level NPs in drinking water following filtration via a nanocomposite membrane.

Experiments conducted in vivo and in vitro reveal that exposure to the nanoparticles investigated results in severe adverse health consequences. In cell-level investigations, exposure to NPs led to cell death, DNA damage, and an increase in reactive oxygen species [305,306]. Due to their small size, NPs may accumulate within cells and may release ions that can directly alter cell function [306]. Animal research conducted in vivo has investigated the effects of NP intake. After 5 days of eating TiO_2_ NPs in drinking water, rats exhibited DNA damage, according to a study [307]. In another study, rats and mice who were fed silver or TiO_2_ nanoparticles had a larger quantity of silver or TiO_2_ in their blood, kidneys, liver, and brain compared to the control group [308,309]. In research, the concentration of Ag ions decreased throughout filtration, and substantial leaching was seen at the start of filtering. However, this quantity dropped as filtering continued [310]. The effects of these increased metal weights are not yet fully understood. Nonetheless, the results suggest that exposure to NPs through digestion might result in the transport of NPs or metal ions throughout the body.

It is essential to design products that do not release nanoparticles into purified water since this poses extra concerns to public health. In order to ensure public health, it is also important to determine if present drinking water treatment procedures properly remove these dangerous chemicals. Despite the vast majority of good and transformational applications of NPs, further research is necessary to keep up with the rapid growth and extension of the potential dangers of this invention. We must come up with a method that assures public health without producing harmful side effects [311].

To verify the non-toxicity assumptions of nanocomposite membranes, more research and a risk–benefit analysis are necessary. Using polymers or natural resources in the production of nanocomposite membranes does not provide a sustainability certification that is guaranteed. The use of nanoparticles in biopolymeric matrices is a serious cause for worry. As predicted, these nanoparticles exert their impact on the nanocomposite membranes, making them high-performing yet less benign. This is the source of the issue, and paradoxically, no studies have confirmed the possible toxicity of nanocomposite membranes using nanoparticles for water purification. Typically, the removal efficiency of contaminants receives the lion’s share of attention, whereas the adverse consequences of nanoparticles are neglected. The likely leaching of these persistent, insoluble nanoparticles into water and, subsequently, their infiltration into the soil [312,313], is another concern that must be addressed.

## 10. Prospective Studies

Despite significant developments in nanocomposite membrane fabrication, additional study is required to address current obstacles. We still have a lot to learn about nanoscale processes involving nanoparticles in membranes, for starters. Incorporating several nanoparticles or multi-element nanostructures into a single polymer matrix has the potential to improve membranes’ long-term stability and performance by offsetting the weaknesses of the components used. The ability to selectively remove pollutants from source water makes metal–organic frameworks (MOFs) an appealing nanofiller [314,315,316], since the availability of alternative water sources for desalination and the development of novel applications for filtrate have increased the value of specialised membrane synthesis, the latter being given preference. Second, nanocomposite membrane development is stymied by the difficulty of preventing nanoparticle aggregation. The current options, which involve surface modification and improvement of production techniques, only apply to certain nanoparticles and polymer matrices. In addition to improving the desirable features of nanocomposite membranes, a standardised approach to better dispersing nanoparticles in the polymer matrix may make the membrane technology more viable for industrial usage. Lastly, more research is needed to determine how nanocomposite membranes hold up over time. Some carbon-based nanoparticles have been found to shield polyamide membranes from chlorine assaults, although the nature of their direct interactions with free chlorine is poorly understood. To identify the effects that prolonged exposure to chlorine has on the functional groups of nanoparticles, there is a pressing need for more study in this area. In addition, there is an absence of exhaustive studies on the processes that are responsible for the release of nanoparticles over polymer membranes. In conclusion, the findings of the study indicate that effective applications of nanocomposite membranes are mostly limited to low-volume environments such as laboratories. The large-scale fabrication technology and long-term testing that are essential for extensive industrial application are currently unavailable.

## 11. Conclusions

The development of hybrid polymer/inorganic nanofiller membranes has led to substantial advancements around polymeric nanocomposite membranes for the treatment of contaminated water, as a consequence. These membranes combine the best properties of inorganic nanofillers, such as photocatalytic degradation capacity, bactericide properties, or absorption capacity, with the improved mechanical, absorption, and chemical properties of polymer matrices. This combination is a critical factor in the broad range of applications described for these hybrid membranes, as it combines the best properties of two different types of materials, including food technology, gas separation, wastewater, desalination, biotechnology, the dairy sector, and the pharmaceutical business. Membrane technology offers medicinal uses such as controlled medication administration, artificial lungs, artificial kidneys, diagnostics for different disorders, and microimaging. Membrane technology also has uses in environmental protection, such as the safe generation of electricity in fuel cells.

The potential for MOFs to improve mass transport inside membranes is becoming an increasingly popular research topic. Metallic nanoparticles have the most significant impact on enhancing heat transfer in nanocomposite membranes, while carbon-based nanoparticles introduced into polymer matrices raise the charge conductivity of the membrane.

The functionality of nanocomposite membranes is superior to that of their bare membrane analogues. The characteristics of the nanoparticles, such as their size and shape, surface qualities, composition, and the kind of polymers that were utilised, have a significant impact on the end result of the performance of nanocomposite membranes. There appears to be an optimum concentration of nanoparticles and polymeric membrane materials for each possible combination, as suggested by the literature. There is no correlation between nanofiller concentration and performance enhancement. Thus, in order to attain the best result, certain combinations of nanoparticles and polymeric membranes require exhaustive experimental assessments.

Despite the abundance of literature, most experiments on nanocomposite membranes have only been conducted for a few months at most. Nanocomposite membranes’ durability and efficacy in the long run are still up for debate. In addition, there has not been a lot of research on how to make nanocomposite membranes on a larger scale. The widespread use of nanocomposite membranes will be considerably facilitated by the development of a mechanistic understanding of nanoparticle polymer matrix interactions and the scaling up of their production. More work needs to be conducted in the future to build stable, high-performance, and scalable nanocomposite membranes for many uses.

The so-called “Valley of Death” is the sometimes-lethal period between a researcher’s scientific findings and the first money required for prototyping and commercialisation. Scientists may publish their findings without being interested in commercialisation. This is a significant gap between research and commercialisation that government organisations and venture investors must overcome.

The inadvertent release of nanoparticles into the environment may be attributed in part to both mechanical and photochemical processes. These processes include grinding, cutting, photo and heat degradation, as well as the features of nanocomposite materials. During the process of nanocomposites being broken down, both nanoscale and microscale fragments as well as nanofillers are formed. As a consequence of the breakdown of the nanocomposite, both workers and consumers are at risk of exposure to particles, especially in the airborne or respirable component of the material. Research indicates that nanoparticles may pose a health risk, with the potential danger posed by these particles varying depending on their make-up, size, and several other physicochemical aspects.

It is obvious that the use of nanomaterials has the potential to make future water treatment facilities more effective, efficient, and economical to run, in addition to making them very simple to build in developing countries that have a limited supply of clean water. PNC membranes will make it possible to create inexpensive and ecologically friendly separation processes, and they will also make it easier to create modular systems that will contribute to the advancement of existing technologies. The implementation of PNCs into long-term separation strategies will bring the factors that influence the economy, society, and environment together and integrate them.

## Figures and Tables

**Table 1:** Polymeric nanocomposite membranes of various sorts.

Type	Polymeric Phase	Nanophase	Functionality	Ref.
Type I: Conventional PNC	Polyvinyl alcohol (PVA) and chitosan	Cellulose nanocrystals/ZnO	Enhanced mechanical characteristics	[101]
	PVA	Nano fibrillated cellulose	The enhancement of mechanical, thermal, and chemical qualities	[102]
		Phosphorylated nanocellulose fibrils	Mechanical, thermal, and chemical characteristics are enhanced	[103]
		Cellulose nanocrystals	Enhancement in tensile strength, thermal stability, and swelling capacity	[104]
	Nafion^@^	Zirconium phosphates + carbon nanotubes	Enhanced mechanical characteristics	[105]
		Zirconium phosphates		[106]
Type II: Active-bulk phase PNC	PVA crosslinked with glutaraldehyde	Nanostructured Fe_3_O_4_/polystyrene core-shell	Adjusting membrane permeability in response to temperature	[108]
	Poly(acrylamide)	Graphene oxide and reduced graphene oxide	Improve thermal conductivity	[109]
	Poly(aniline)	Graphene oxide	Sensitive to NH_3_ gas	[110]
	Conductive poly(vinylidene fluoride)	Carbon nanofiber + ionic liquid	Sensitive to strain	[111]
	Cellulose	Copper Oxide	Gaseous compound detection at low temperatures (e.g., H_2_S)	[112]
Type III: Active-surface PNC	Aromatic polyamide	Carbon nanotubes	Antifouling, increased permeability, and chlorine resistance	[113]
	Polyethersulfone (PES)	Fe_2_O_3_ nanoparticles	Ionic strength sensitive	[114]
		Carboxylated graphene	Anti-biofouling properties and protein antifouling	[116]
	Polyamide (PA)	Natural zeolite nanoparticles	Nitrate rejection and permeability increase	[115]
		Highly hydrophilic clay mineral + Ag	Increased NaCl rejection and anti-biofouling	[117]
		Silica nanoparticles functionalised with quaternary ammonium groups	Super hydrophilic, antifouling, and very permeable to water	[118]

**Table 2:** Some potential applications for composite nanofibrous membranes that are supported by polymeric matrices in the field of water purification.

Nanocomponent	Membrane Composite System	Application	Ref.
TiO_2_ NPs	poly(vinylpyrrolidone) (PVP)-co-poly (vinylidene fluoride) (PVDF)/P_25_-TiO_2_ NPs	Organic pollutants are broken down by photocatalysis	[177]
	poly(ethersulfones) (PES)/TiO_2_ NPs/water soluble porphyrins	Heavy metals are easy to find and remove	[178]
Carbon Nanotubes (CNT)	poly(acrylonitrile)(PAN)/CNT/TiO_2_-NH_2_ NPs	Metal ions can be broken down by photocatalysis	[179]
	PES/single-walled CNT (SWCNTs)	Membranes with antimicrobial activity	[180]
	poly(vinyl alcohol) (PVA)/bovine serum albumin (BSA)/SWNTs	There was more enzyme binding and ester hydrolysis	[181]
SiO_2_ NPs	PVDF/SiO_2_ NPs	Increase in flux (24 Lm^−2^·h^−1^) in water–oil separations	[182]
	PVDF/Amorphous SiO_2_ NPs	Forward osmosis (FO) desalination showed an increase in water flow (83 Lm^−2^·h^−1^)	[183]
	PVDF/SiO_2_ NPs	For processes of membrane distillation (MD)	[184]
Ag NPs	Cellulose acetate/Ag NPs	Antimicrobial activity	[185]
	poly(lactide-co-glycoside) PLGA/chitosan (10%)/graphene-oxide-Ag decorated NPs (GO-Ag NPs)	Antimicrobial activity	[186]
	Polycaprolactone (PCL)/TiO_2_-Ag NPs	Photocatalytic and antibacterial activity membranes	[187]
ZnO	Nylon 6,6/ZnO core-shell nanofibers	Photocatalytic membranes that are very stable and flexible	[188]
	Polyurethane (PU)/polydopamine/ZnO nanorods	Antifouling membranes that work through photocatalysis	[189]
	PAN/ZnO-Ag heterostructure NPs	Antibacterial activity	[190]
Fe NPs	Poly(acrylic acid) (PAA)/polyvinyl alcohol (PVA)/FeCl_3_ (aq.)	Complexation of Fe^3+^ ions and creation of new Fe nanoparticles (NPs), dye degradation	[191]
Fe_2_O_3_ NPs	poly(lactic acid)/γ-Fe_2_O_3_ NPs	Oil is absorbed and separated very efficiently	[192]
Yeast Cells	Core: PVP/Yeast Cells Sheath: PVDF-co-hexafluoropropylene (HFP)/poly(ethylene glycol) (PEG)	Degradation of phenol	[193]

**Table 3:** Comparison of the key benefits and drawbacks of the various membrane production procedures.

Fabrication Technique	Key Benefits	Drawbacks
Interfacial polymerisation	Low cost, high simplicity, and reproducibility Pore size uniformity Excellent antifouling qualities	Low flux High retention
Phase inversion	Stability Film formation is quick Simplicity and repeatability High flux	Non-uniform pore size Limited materials
Track-etching	Structure subject to control Pore size uniformity High reproducibility Exact structure	High cost Low simplicity Low flux
Electrospinning	High flux Mass production	Non-uniform pore size Medium cost Solvent required Low mechanical stability
3D printing	Cost effective Complex and tightly regulated structures Flexibility Stability both chemically and mechanically	Limited materials Size limitations Post-processing time

## Data Availability

Not applicable.

## References

[1] Drinking-Water. https://www.who.int/news-room/fact-sheets/detail/drinking-water.

[2] Pendergast M.M., Hoek E.M.V. (2011). A review of water treatment membrane nanotechnologies. Energy Environ. Sci..

[3] Arias-Estévez M., López-Periago E., Martínez-Carballo E., Simal-Gándara J., Mejuto J.C., García-Río L. (2008). The mobility and degradation of pesticides in soils and the pollution of groundwater resources. Agric. Ecosyst. Environ..

[4] Lin W.C., Li Z., Burns M.A. (2017). A Drinking Water Sensor for Lead and Other Heavy Metals. Anal. Chem..

[5] Hunt H., Briscoe H.T. (1929). The electrical conductivity of organic acids in water, alcohols, and acetone, and the electronic structures of the acids. J. Phys. Chem..

[6] Kremser U., Schnug E. (2002). Impact of fertilizers on aquatic ecosystems and protection of water bodies from mineral nutrients. Landbauforsch. Volkenrode.

[7] Naim M.M., Al-Harby N.F., El Batouti M., Elewa M.M. (2022). Macro-Reticular Ion Exchange Resins for Recovery of Direct Dyes from Spent Dyeing and Soaping Liquors. Molecules.

[8] Nabeela F., Azizullah A., Bibi R., Uzma S., Murad W., Shakir S.K., Ullah W., Qasim M., Häder D.P. (2014). Microbial contamination of drinking water in Pakistan—A review. Environ. Sci. Pollut. Res..

[9] El Batouti M., Al-Harby N.F., Elewa M.M. (2021). A Review on Promising Membrane Technology Approaches for Heavy Metal Removal from Water and Wastewater to Solve Water Crisis. Water.

[10] Ali S., Rehman S.A.U., Shah I.A., Farid M.U., An A.K., Huang H. (2019). Efficient removal of zinc from water and wastewater effluents by hydroxylated and carboxylated carbon nanotube membranes: Behaviors and mechanisms of dynamic filtration. J. Hazard. Mater..

[11] Masood N., Farooqi A., Zafar M.I. (2019). Health risk assessment of arsenic and other potentially toxic elements in drinking water from an industrial zone of Gujrat, Pakistan: A case study. Environ. Monit. Assess..

[12] Pandey N., Shukla S.K., Singh N.B. (2017). Water purification by polymer nanocomposites: An overview. Nanocomposites.

[13] Sharma A., Tulsyan A., Motamarri S. (2009). A comparative study of chromium (VI) removal using sawdust and eucalyptus bark. Water Sci. Technol. Water Supply.

[14] Yin J., Deng B. (2015). Polymer-matrix nanocomposite membranes for water treatment. J. Membr. Sci..

[15] Shannon M.A., Bohn P.W., Elimelech M., Georgiadis J.G., Mariñas B.J., Mayes A.M., Marĩas B.J., Mayes A.M. (2008). Science and technology for water purification in the coming decades. Nature.

[16] Qadir D., Mukhtar H., Keong L.K. (2017). Mixed Matrix Membranes for Water Purification Applications. Sep. Purif. Rev..

[17] Baig N., Salhi B., Sajid M., Aljundi I.H. (2022). Recent Progress in Microfiltration/Ultrafiltration Membranes for Separation of Oil and Water Emulsions. Chem. Rec..

[18] Jiang J., Ma B., Yang C., Duan X., Tang Q. (2022). Fabrication of anti-fouling and photocleaning PVDF microfiltration membranes embedded with N-TiO_2_ photocatalysts. Sep. Purif. Technol..

[19] Antolín-Cerón V.H., González-López F.J., Astudillo-Sánchez P.D., Barrera-Rivera K.A., Martínez-Richa A. (2022). High-Performance Polyurethane Nanocomposite Membranes Containing Cellulose Nanocrystals for Protein Separation. Polymer.

[20] Yousef S., Eimontas J., Striūgas N., Mohamed A., Ali Abdelnaby M. (2022). Pyrolysis kinetic behavior and TG-FTIR-GC–MS analysis of end-life ultrafiltration polymer nanocomposite membranes. Chem. Eng. J..

[21] Naim M.M., El-Shafei A.A., Moneer A.A., Elewa M.M. (2015). Ultrafiltration by a super-hydrophilic regenerated cellulose membrane. Water Pract. Technol..

[22] Khoerunnisa F., Nurhayati M., Annisa N.A.A., Fatimah S., Nashrah N., Hendrawan H., Ko Y.G., Ng E.P., Opaprakasit P. (2022). Effects of Benzalkonium Chloride Contents on Structures, Properties, and Ultrafiltration Performances of Chitosan-Based Nanocomposite Membranes. Membranes.

[23] Li S., Yin Y., Liu S., Li H., Su B., Han L., Gao X., Gao C. (2022). Interlayered thin-film nanocomposite membrane with synergetic effect of COFs interlayer and GQDs incorporation for organic solvent nanofiltration. J. Membr. Sci..

[24] Dashtbozorg A., Saljoughi E., Mousavi S.M., Kiani S. (2022). High-performance and robust polysulfone nanocomposite membrane containing 2D functionalized MXene nanosheets for the nanofiltration of salt and dye solutions. Desalination.

[25] Zhang W., Xu H., Xie F., Ma X., Niu B., Chen M., Zhang H., Zhang Y., Long D. (2022). General synthesis of ultrafine metal oxide/reduced graphene oxide nanocomposites for ultrahigh-flux nanofiltration membrane. Nat. Commun..

[26] Liu Y., Wang X.-P., Zong Z.-A., Lin R., Zhang X.-Y., Chen F.-S., Ding W.-D., Zhang L.-L., Meng X.-M., Hou J. (2022). Thin film nanocomposite membrane incorporated with 2D-MOF nanosheets for highly efficient reverse osmosis desalination. J. Membr. Sci..

[27] Bian S., Wang Y., Xiao F., Tong Y., Gao C., Zhu G. (2022). Fabrication of polyamide thin-film nanocomposite reverse osmosis membrane with improved permeability and antibacterial performances using silver immobilized hollow polymer nanospheres. Desalination.

[28] Zhao Q., Zhao D.L., Feng F., Chung T.S., Chen S.B. (2022). Thin-film nanocomposite reverse osmosis membranes incorporated with citrate-modified layered double hydroxides (LDHs) for brackish water desalination and boron removal. Desalination.

[29] Burts K., Plisko T., Dmitrenko M., Zolotarev A., Kuzminova A., Bildyukevich A., Ermakov S., Penkova A. (2022). Novel Thin Film Nanocomposite Membranes Based on Chitosan Succinate Modified with Fe-BTC for Enhanced Pervaporation Dehydration of Isopropanol. Membranes.

[30] Elewa M.M., El-Shafei A.A., Moneer A.A., Naim M.M. (2016). Effect of cell hydrodynamics in desalination of saline water by sweeping air pervaporation technique using innovated membrane. Desalin. Water Treat..

[31] Naim M., Elewa M., El-Shafei A., Moneer A. (2015). Desalination of simulated seawater by purge-air pervaporation using an innovative fabricated membrane. Water Sci. Technol..

[32] Elsheniti M.B., Elbessomy M.O., Wagdy K., Elsamni O.A., Elewa M.M. (2021). Augmenting the distillate water flux of sweeping gas membrane distillation using turbulators: A numerical investigation. Case Stud. Therm. Eng..

[33] Yadav A., Mandal J.R., Panda A.B., Shahi V.K. (2022). Structural tailoring of ceria nanoparticles for fabricating fouling resistant nanocomposite membranes with high flux distillation. Colloids Surf. A Physicochem. Eng. Asp..

[34] Gontarek-Castro E., Castro-Muñoz R., Lieder M. (2022). New insights of nanomaterials usage toward superhydrophobic membranes for water desalination via membrane distillation: A review. Crit. Rev. Environ. Sci. Technol..

[35] Yassari M., Shakeri A., Salehi H., Razavi S.R. (2022). Enhancement in forward osmosis performance of thin-film nanocomposite membrane using tannic acid-functionalized graphene oxide. J. Polym. Res..

[36] Meier-Haack J. (2022). Special Issue: New Challenges in Thin-Film Nanocomposite Membranes. Coatings.

[37] Wei X., Liu Y., Zheng J., Wang X., Xia S., Van der Bruggen B. (2022). A critical review on thin-film nanocomposite membranes enabled by nanomaterials incorporated in different positions and with diverse dimensions: Performance comparison and mechanisms. J. Membr. Sci..

[38] Bernardo P., Drioli E., Golemme G. (2009). Membrane gas separation: A review/state of the art. Ind. Eng. Chem. Res..

[39] Ulbricht M. (2006). Advanced functional polymer membranes. Polymer.

[40] Goh P.S., Ismail A.F. (2018). A review on inorganic membranes for desalination and wastewater treatment. Desalination.

[41] Ng L.Y., Mohammad A.W., Leo C.P., Hilal N. (2013). Polymeric membranes incorporated with metal/metal oxide nanoparticles: A comprehensive review. Desalination.

[42] Lee A., Elam J.W., Darling S.B. (2016). Membrane materials for water purification: Design, development, and application. Environ. Sci. Water Res. Technol..

[43] Lee J., Jeong S., Liu Z. (2016). Progress and challenges of carbon nanotube membrane in water treatment. Crit. Rev. Environ. Sci. Technol..

[44] Lee J., Ye Y., Ward A.J., Zhou C., Chen V., Minett A.I., Lee S., Liu Z., Chae S.R., Shi J. (2016). High flux and high selectivity carbon nanotube composite membranes for natural organic matter removal. Sep. Purif. Technol..

[45] El Batouti M., Alharby N.F., Elewa M.M. (2021). Review of New Approaches for Fouling Mitigation in Membrane Separation Processes in Water Treatment Applications. Separations.

[46] Raval H.D., Trivedi J.J., Joshi S.V., Devmurari C.V. (2010). Flux enhancement of thin film composite RO membrane by controlled chlorine treatment. Desalination.

[47] Guo W., Ngo H.-H.H., Li J. (2012). A mini-review on membrane fouling. Bioresour. Technol..

[48] Hegab H.M., Zou L. (2015). Graphene oxide-assisted membranes: Fabrication and potential applications in desalination and water purification. J. Membr. Sci..

[49] Li X., Janke A., Formanek P., Fery A., Stamm M., Tripathi B.P. (2018). One pot preparation of polysulfone-amino functionalized SiO_2_ nanoparticle ultrafiltration membranes for water purification. J. Environ. Chem. Eng..

[50] Esfahani M.R., Tyler J.L., Stretz H.A., Wells M.J.M. (2015). Effects of a dual nanofiller, nano-TiO_2_ and MWCNT, for polysulfone-based nanocomposite membranes for water purification. Desalination.

[51] Zhang L., Shan C., Jiang X., Li X., Yu L. (2018). High hydrophilic antifouling membrane modified with capsaicin-mimic moieties via microwave assistance (MWA) for efficient water purification. Chem. Eng. J..

[52] Vatanpour V., Jouyandeh M., Akhi H., Mousavi Khadem S.S., Ganjali M.R., Moradi H., Mirsadeghi S., Badiei A., Esmaeili A., Rabiee N. (2022). Hyperbranched polyethylenimine functionalized silica/polysulfone nanocomposite membranes for water purification. Chemosphere.

[53] Barzegar T., Hassanajili S. (2022). Fabrication and characterization of dual layer PEBAX-SiO_2_/polyethersulfone nanocomposite membranes for separation of CO_2_/CH_4_ gases. J. Appl. Polym. Sci..

[54] Yoon K., Hsiao B.S., Chu B. (2009). High flux ultrafiltration nanofibrous membranes based on polyacrylonitrile electrospun scaffolds and crosslinked polyvinyl alcohol coating. J. Membr. Sci..

[55] Iranpoury A., Mehrnia M.R., Jafari S.H., Najmi M. (2022). Improvement of fouling resistance and mechanical reinforcement of polyacrylonitrile membranes by amino-functionalized multiwalled carbon nanotubes for membrane bioreactors applications. J. Appl. Polym. Sci..

[56] Tan Z., Chen S., Peng X., Zhang L., Gao C. (2018). Polyamide membranes with nanoscale Turing structures for water purification. Science.

[57] Vatanpour V., Iranpour Boroujeni N., Pasaoglu M.E., Mahmodi G., Mohammadikish M., Kazemi-Andalib F., Koyuncu I. (2022). Novel infinite coordination polymer (ICP) modified thin-film polyamide nanocomposite membranes for simultaneous enhancement of antifouling and chlorine-resistance performance. J. Membr. Sci..

[58] Muhammad S., Niazi J.H., Shawuti S., Qureshi A. (2022). Functional POSS based polyimide nanocomposite for enhanced structural, thermal, antifouling and antibacterial properties. Mater. Today Commun..

[59] Li E., Chen Z., Duan C., Yuan B., Yan S., Luo X., Pan F., Jiang Z. (2022). Enhanced CO_2_-capture performance of polyimide-based mixed matrix membranes by incorporating ZnO@MOF nanocomposites. Sep. Purif. Technol..

[60] Nouri M., Marjani A., Tajdari M., Heidary F., Salimi M. (2018). Preparation of cellulose acetate membrane coated by PVA/Fe_3_O_4_ nanocomposite thin film: An in situ procedure. Colloid Polym. Sci..

[61] Bhanthumnavin W., Wanichapichart P., Taweepreeda W., Sirijarukula S., Paosawatyanyong B. (2016). Surface modification of bacterial cellulose membrane by oxygen plasma treatment. Surf. Coat. Technol..

[62] Keskin B., Naziri Mehrabani S.A., Arefi-Oskoui S., Vatanpour V., Orhun Teber O., Khataee A., Orooji Y., Koyuncu I. (2022). Development of Ti_2_AlN MAX phase/cellulose acetate nanocomposite membrane for removal of dye, protein and lead ions. Carbohydr. Polym..

[63] Elbadawi N.A., Ramadan A.R., Esawi A.M.K. (2022). Studying the Effect of Shortening Carbon Nanotubes via Ball Milling on Cellulose Acetate Nanocomposite Membranes for Desalination Applications. Membranes.

[64] Gao A., Liu F., Xue L. (2014). Preparation and evaluation of heparin-immobilized poly (lactic acid) (PLA) membrane for hemodialysis. J. Membr. Sci..

[65] Vatanpour V., Dehqan A., Paziresh S., Zinadini S., Zinatizadeh A.A., Koyuncu I. (2022). Polylactic acid in the fabrication of separation membranes: A review. Sep. Purif. Technol..

[66] Yakdoumi F.Z., Hadj-Hamou A.S., Rahoui N., Rahman M.M., Abetz V. (2022). Polylactic acid nanocomposites containing functionalized multiwalled carbon nanotubes as antimicrobial packaging materials. Int. J. Biol. Macromol..

[67] He Y., Yan J., He X., Weng W., Cheng K. (2022). PLLA/Graphene Nanocomposites Membranes with Improved Biocompatibility and Mechanical Properties. Coatings.

[68] Svang-Ariyaskul A., Huang R.Y.M., Douglas P.L., Pal R., Feng X., Chen P., Liu L. (2006). Blended chitosan and polyvinyl alcohol membranes for the pervaporation dehydration of isopropanol. J. Membr. Sci..

[69] Ahmad S., Jahan Z., Sher F., Niazi M.B.K., Noor T., Hou H., Azhar O., Sher E.K. (2022). Polyvinyl alcohol and aminated cellulose nanocrystal membranes with improved interfacial compatibility for environmental applications. Environ. Res..

[70] Asadpour S., Raeisi vanani A., Kooravand M., Asfaram A. (2022). A review on zinc oxide/poly(vinyl alcohol) nanocomposites: Synthesis, characterization and applications. J. Clean. Prod..

[71] Liu F., Hashim N.A., Liu Y., Abed M.R.M., Li K. (2011). Progress in the production and modification of PVDF membranes. J. Membr. Sci..

[72] Tomaszewska M. (1996). Preparation and properties of flat-sheet membranes from poly(vinylidene fluoride) for membrane distillation. Desalination.

[73] Pagliero M., Alloisio M., Costa C., Firpo R., Mideksa E.A., Comite A. (2022). Carbon Black/Polyvinylidene Fluoride Nanocomposite Membranes for Direct Solar Distillation. Energies.

[74] Kumar A., Ghosh U.K. (2022). Polyvinylidene fluoride/boehmite nanocomposite membrane for effective removal of arsenate ion from water. J. Water Process Eng..

[75] Chai J., Wang G., Zhang A., Dong G., Li S., Zhao J., Zhao G. (2022). Microcellular injection molded lightweight and tough poly (L-lactic acid)/in-situ polytetrafluoroethylene nanocomposite foams with enhanced surface quality and thermally-insulating performance. Int. J. Biol. Macromol..

[76] Asadi A., Gholami F., Nazari S., Dolatshah M. (2022). Improved filtration performance of polyvinylidene fluoride nanocomposite membranes embedded with deep eutectic solvent: Application towards MBR. Desalination.

[77] Sun D., Zhou T., Lu Y., Yan Y., Liu C., Che G. (2022). Ion-imprinted antifouling nanocomposite membrane for separation of lithium ion. Korean J. Chem. Eng..

[78] Observatory Nano (2011). Nanoenhanced Membranes for Improved Water Treatment Briefing No.16 Environment.

[79] Buonomenna M.G. (2013). Nano-enhanced reverse osmosis membranes. Desalination.

[80] Chen X., Gao X., Fu K., Qiu M., Xiong F., Ding D., Cui Z., Wang Z., Fan Y., Drioli E. (2018). Tubular hydrophobic ceramic membrane with asymmetric structure for water desalination via vacuum membrane distillation process. Desalination.

[81] Castro-Muñoz R., Galiano F., Fíla V., Drioli E., Figoli A. (2018). Matrimid^®^5218 dense membrane for the separation of azeotropic MeOH-MTBE mixtures by pervaporation. Sep. Purif. Technol..

[82] Ursino C., Castro-Muñoz R., Drioli E., Gzara L., Albeirutty M.H., Figoli A. (2018). Progress of nanocomposite membranes for water treatment. Membranes.

[83] Zoromba M.S., Ismail M.I.M., Bassyouni M.I., Abdel-Aziz M.H., Salah N., Alshahrie A., Memic A. (2017). Fabrication and characterization of poly (aniline-co-o-anthranilic acid)/magnetite nanocomposites and their application in wastewater treatment. Colloids Surf. A Physicochem. Eng. Asp..

[84] Misdan N., Ismail A.F., Hilal N. (2016). Recent advances in the development of (bio) fouling resistant thin film composite membranes for desalination. Desalination.

[85] Cui L., Chen P., Chen S., Yuan Z., Yu C., Ren B., Zhang K. (2013). In situ study of the antibacterial activity and mechanism of action of silver nanoparticles by surface-enhanced raman spectroscopy. Anal. Chem..

[86] Li J., Wang G., Shi Z., Yang M., Luck R.L. (2009). Two hydrogen-bond-cross-linked molybdenum (VI) network polymers: Synthesis, crystal structures and cyclooctene epoxidation with H_2_O_2_. Struct. Chem..

[87] Marambio-Jones C., Hoek E.M.V. (2010). A review of the antibacterial effects of silver nanomaterials and potential implications for human health and the environment. J. Nanopart. Res..

[88] Naim M.M., El-Shafei A.A., Elewa M.M., Moneer A.A. (2017). Application of silver-, iron-, and chitosan- nanoparticles in wastewater treatment. Desalin. Water Treat..

[89] Li X., Sotto A., Li J., Van der Bruggen B. (2017). Progress and perspectives for synthesis of sustainable antifouling composite membranes containing in situ generated nanoparticles. J. Membr. Sci..

[90] Gholami A., Moghadassi A.R., Hosseini S.M., Shabani S., Gholami F. (2014). Preparation and characterization of polyvinyl chloride based nanocomposite nanofiltration-membrane modified by iron oxide nanoparticles for lead removal from water. J. Ind. Eng. Chem..

[91] Hernandez M., Medioni G., Hu Z., Sadda S. Multimodal registration of multiple retinal images based on line structures. Proceedings of the 2015 IEEE Winter Conference on Applications of Computer Vision.

[92] Huang Q., Shi X., Pinto R.A., Petersen E.J., Weber W.J. (2008). Tunable synthesis and immobilization of zero-valent iron nanoparticles for environmental applications. Environ. Sci. Technol..

[93] Ahmad R., Sharma N., Mishra C., Singh N.J., Rawat G.S., Bhatnagar Y.V. (2018). Security, size, or sociality: What makes markhor (Capra falconeri) sexually segregate?. J. Mammal..

[94] Mastropietro T.F., Drioli E., Poerio T. (2014). Low temperature synthesis of nanosized NaY zeolite crystals from organic-free gel by using supported seeds. RSC Adv..

[95] Gutub S.A., Bassyouni M., Abdel-Hamid S.M.S. (2013). Dissolved solids adsorption of freshwater using synthesized bio-foam composite. Life Sci. J..

[96] Dasgupta J., Chakraborty S., Sikder J., Kumar R., Pal D., Curcio S., Drioli E. (2014). The effects of thermally stable titanium silicon oxide nanoparticles on structure and performance of cellulose acetate ultrafiltration membranes. Sep. Purif. Technol..

[97] Alsalhy Q.F., Salih H.A., Simone S., Zablouk M., Drioli E., Figoli A. (2014). Poly (ether sulfone) (PES) hollow-fiber membranes prepared from various spinning parameters. Desalination.

[98] Rizzuto C., Pugliese G., Bahattab M.A., Aljlil S.A., Drioli E., Tocci E. (2018). Multiwalled carbon nanotube membranes for water purification. Sep. Purif. Technol..

[99] Chen B., Sun W., Wang C., Guo X. (2017). Size-dependent impact of inorganic nanoparticles on sulfamethoxazole adsorption by carbon nanotubes. Chem. Eng. J..

[100] Kaur H., Bulasara V.K., Gupta R.K. (2016). Effect of carbonates composition on the permeation characteristics of low-cost ceramic membrane supports. J. Ind. Eng. Chem..

[101] Azizi S., Ahmad M.B., Ibrahim N.A., Hussein M.Z., Namvar F. (2014). Cellulose nanocrystals/ZnO as a bifunctional reinforcing nanocomposite for poly(vinyl alcohol)/chitosan blend films: Fabrication, characterization and properties. Int. J. Mol. Sci..

[102] Poyraz B., Tozluoğlu A., Candan Z., Demir A., Yavuz M. (2017). Influence of PVA and silica on chemical, thermo-mechanical and electrical properties of Celluclast-treated nanofibrillated cellulose composites. Int. J. Biol. Macromol..

[103] Niazi M.B.K., Jahan Z., Berg S.S., Gregersen Ø.W. (2017). Mechanical, thermal and swelling properties of phosphorylated nanocellulose fibrils/PVA nanocomposite membranes. Carbohydr. Polym..

[104] Jahan Z., Niazi M.B.K., Gregersen Ø.W. (2018). Mechanical, thermal and swelling properties of cellulose nanocrystals/PVA nanocomposites membranes. J. Ind. Eng. Chem..

[105] Sigwadi R., Dhlamini M.S., Mokrani T., Nemavhola F. (2019). Enhancing the mechanical properties of zirconia/Nafion^®^ nanocomposite membrane through carbon nanotubes for fuel cell application. Heliyon.

[106] Sigwadi R., Dhlamini M.S., Mokrani T., Ṋemavhola F., Nonjola P.F., Msomi P.F. (2019). The proton conductivity and mechanical properties of Nafion^®^/ZrP nanocomposite membrane. Heliyon.

[107] Naim M.M., Batouti M.E., Elewa M.M. (2021). Novel heterogeneous cellulose-based ion-exchange membranes for electrodialysis. Polym. Bull..

[108] Csetneki I., Filipcsei G., Zrínyi M. (2006). Smart nanocomposite polymer membranes with on/off switching control. Macromolecules.

[109] Shemshadi R., Ghafarian R., Gorji M., Avazverdi E. (2018). A smart thermoregulatory nanocomposite membrane with improved thermal properties: Simultaneous use of graphene family and micro-encapsulated phase change material. Text. Res. J..

[110] Lee C.T., Wang Y.S. (2019). High-performance room temperature NH_3_ gas sensors based on polyaniline-reduced graphene oxide nanocomposite sensitive membrane. J. Alloys Compd..

[111] Prasad B., Gill F.S., Panwar V., Anoop G. (2019). Development of strain sensor using conductive poly(vinylidene fluoride) (PVDF) nanocomposite membrane reinforced with ionic liquid (IL) & carbon nanofiber (CNF). Compos. Part B Eng..

[112] Hittini W., Abu-Hani A.F., Reddy N., Mahmoud S.T. (2020). Cellulose-Copper Oxide hybrid nanocomposites membranes for H_2_S gas detection at low temperatures. Sci. Rep..

[113] Inukai S., Cruz-Silva R., Ortiz-Medina J., Morelos-Gomez A., Takeuchi K., Hayashi T., Tanioka A., Araki T., Tejima S., Noguchi T. (2015). High-performance multi-functional reverse osmosis membranes obtained by carbon nanotube·polyamide nanocomposite. Sci. Rep..

[114] Emami N., Razmjou A., Noorisafa F., Korayem A.H., Zarrabi A., Ji C. (2017). Fabrication of smart magnetic nanocomposite asymmetric membrane capsules for the controlled release of nitrate. Environ. Nanotechnol. Monit. Manag..

[115] Ghaee A., Zerafat M.M., Askari P., Sabbaghi S., Sadatnia B. (2017). Fabrication of polyamide thin-film nanocomposite membranes with enhanced surface charge for nitrate ion removal from water resources. Environ. Technol..

[116] Shukla A.K., Alam J., Ansari M.A., Alhoshan M., Ali F.A.A. (2018). Antimicrobial and antifouling properties of versatile PPSU/carboxylated GO nanocomposite membrane against Gram-positive and Gram-negative bacteria and protein. Environ. Sci. Pollut. Res..

[117] Wang W., Li Y., Wang W., Gao B., Wang Z. (2019). Palygorskite/silver nanoparticles incorporated polyamide thin film nanocomposite membranes with enhanced water permeating, antifouling and antimicrobial performance. Chemosphere.

[118] Shakeri A., Salehi H., Ghorbani F., Amini M., Naslhajian H. (2019). Polyoxometalate based thin film nanocomposite forward osmosis membrane: Superhydrophilic, anti-fouling, and high water permeable. J. Colloid Interface Sci..

[119] Wen Y., Yuan J., Ma X., Wang S., Liu Y. (2019). Polymeric nanocomposite membranes for water treatment: A review. Environ. Chem. Lett..

[120] Palencia M., Córdoba A., Vera M. (2016). Membrane Technology and Chemistry. Nanostruct. Polym. Membr..

[121] Palencia M., Martínez-Lara J.M., Chate-Galvis N.G., Durango-Petro J.M., Shalan A.E., Hamdy Makhlouf A.S., Lanceros-Méndez S. (2022). Functionality-Structure Relationship into Functional Polymeric Nanocomposite Membranes for Removal and Monitoring of Pollutants in Fluid Phases. Engineering Materials.

[122] Cong H., Radosz M., Towler B.F., Shen Y. (2007). Polymer-inorganic nanocomposite membranes for gas separation. Sep. Purif. Technol..

[123] Pourzare K., Mansourpanah Y., Farhadi S. (2016). Advanced nanocomposite membranes for fuel cell applications: A comprehensive review. Biofuel Res. J..

[124] Bee S.L., Abdullah M.A.A., Bee S.T., Sin L.T., Rahmat A.R. (2018). Polymer nanocomposites based on silylated-montmorillonite: A review. Prog. Polym. Sci..

[125] Alateyah A.I., Dhakal H.N., Zhang Z.Y. (2013). Processing, properties, and applications of polymer nanocomposites based on layer silicates: A review. Adv. Polym. Technol..

[126] Paul D.R., Robeson L.M. (2008). Polymer nanotechnology: Nanocomposites. Polymer.

[127] Sheikholeslami S.N., Rafizadeh M., Taromi F.A., Bouhendi H. (2014). Synthesis and characterization of poly(trimethylene terephthalate)/organoclay nanocomposite via in situ polymerization: Including thermal properties and dyeability. J. Thermoplast. Compos. Mater..

[128] Alexandre M., Dubois P. (2000). Polymer-layered silicate nanocomposites: Preparation, properties and uses of a new class of materials. Mater. Sci. Eng. R Rep..

[129] Pavlidou S., Papaspyrides C.D. (2008). A review on polymer-layered silicate nanocomposites. Prog. Polym. Sci..

[130] VanderHart D.L., Asano A., Gilman J.W. (2001). Solid-state NMR investigation of paramagnetic nylon-6 clay nanocomposites. 2. Measurement of clay dispersion, crystal stratification, and stability of organic modifiers. Chem. Mater..

[131] Gao F. (2004). Clay/polymer composites: The story. Mater. Today.

[132] Panwar A., Choudhary V., Sharma D.K. (2011). Review: A review: Polystyrene/clay nanocomposites. J. Reinf. Plast. Compos..

[133] Usuki A., Kojima Y., Kawasumi M., Okada A., Fukushima Y., Kurauchi T., Kamigaito O. (1993). Synthesis of nylon 6-clay hybrid. J. Mater. Res..

[134] Doucouré A., Guizard C., Durand J., Berjoan R., Cot L. (1996). Plasma polymerization of fluorinated monomers on mesoporous silica membranes and application to gas permeation. J. Membr. Sci..

[135] Patel N.P., Miller A.C., Spontak R.J. (2003). Highly CO_2_-permeable and selective polymer nanocomposite membranes. Adv. Mater..

[136] Patel N.P., Aberg C.M., Sanchez A.M., Capracotta M.D., Martin J.D., Spontak R.J. (2004). Morphological, mechanical and gas-transport characteristics of crosslinked poly(propylene glycol): Homopolymers, nanocomposites and blends. Polymer.

[137] Nunes S.P., Peinemann K.V., Ohlrogge K., Alpers A., Keller M., Pires A.T.N. (1999). Membranes of poly(ether imide) and nanodispersed silica. J. Membr. Sci..

[138] Rubio L.R., Teijido R., Veloso-Fernández A., Pérez-Yáñez S., Vilas-Vilela J.L., Shalan A.E., Hamdy Makhlouf A.S., Lanceros-Méndez S. (2022). Polymeric Nanocomposite Membranes for Water Remediation: From Classic Approaches to 3D Printing. Engineering Materials.

[139] Bounor-Legaré V., Cassagnau P. (2014). In situ synthesis of organic-inorganic hybrids or nanocomposites from sol-gel chemistry in molten polymers. Prog. Polym. Sci..

[140] Brzesowsky R.H., De With G., Van Den Cruijsem S., Snijkers-Hendrickx I.J.M., Wolter W.A.M., Van Lierop J.G. (1998). Glass strengthening by silica particle reinforced organic-inorganic coatings. J. Non-Cryst. Solids.

[141] Livage J., Sanchez C. (1992). Sol-gel chemistry. J. Non-Cryst. Solids.

[142] Kioul A., Mascia L. (1994). Compatibility of polyimide-silicate ceramers induced by alkoxysilane silane coupling agents. J. Non-Cryst. Solids.

[143] Smaïhi M., Jermoumi T., Marignan J., Noble R.D. (1996). Organic-inorganic gas separation membranes: Preparation and characterization. J. Membr. Sci..

[144] Day V.W., Eberspacher T.A., Chen Y., Hao J., Klemperer W.G. (1995). Low-nuclearity titanium oxoalkoxides: The trititanates [Ti_3_O](OPri)10 and [Ti_3_O](OPri)9(OMe). Inorg. Chim. Acta.

[145] Livage J., Aegerter M.A., Mennig M. (2004). Basic Principles of Sol-Gel Chemistry. Sol-Gel Technologies for Glass Producers and Users.

[146] Iwata M., Adachi T., Tomidokoro M., Ohta M., Kobayashi T. (2003). Hybrid sol-gel membranes of polyacrylonitrile-tetraethoxysilane composites for gas permselectivity. J. Appl. Polym. Sci..

[147] Gomes D., Nunes S.P., Peinemann K.V. (2005). Membranes for gas separation based on poly(1-trimethylsilyl-1-propyne)- silica nanocomposites. J. Membr. Sci..

[148] Guo Y., Wang X., Hu P., Peng X. (2016). ZIF-8 coated polyvinylidenefluoride (PVDF) hollow fiber for highly efficient separation of small dye molecules. Appl. Mater. Today.

[149] Stephen R., Ranganathaiah C., Varghese S., Joseph K., Thomas S. (2006). Gas transport through nano and micro composites of natural rubber (NR) and their blends with carboxylated styrene butadiene rubber (XSBR) latex membranes. Polymer.

[150] Mascia L., Zhang Z., Shaw S.J. (1996). Carbon fibre composites based on polyimide/silica ceramers: Aspects of structure-properties relationship. Compos. Part A Appl. Sci. Manuf..

[151] Gacitua W., Ballerini A., Zhang J. (2005). Polymer Nanocomposites: Synthetic and Natural Fillers a Review. Maderas Cienc. Y Tecnol..

[152] Ragab D., Gomaa H.G., Sabouni R., Salem M., Ren M., Zhu J. (2016). Micropollutants removal from water using microfiltration membrane modified with ZIF-8 metal organic frameworks (MOFs). Chem. Eng. J..

[153] Low Z.X., Razmjou A., Wang K., Gray S., Duke M., Wang H. (2014). Effect of addition of two-dimensional ZIF-L nanoflakes on the properties of polyethersulfone ultrafiltration membrane. J. Membr. Sci..

[154] Bhiwankar N.N., Weiss R.A. (2006). Melt intercalation/exfoliation of polystyrene-sodium-montmorillonite nanocomposites using sulfonated polystyrene ionomer compatibilizers. Polymer.

[155] Yoon J.T., Jo W.H., Lee M.S., Ko M.B. (2001). Effects of comonomers and shear on the melt intercalation of styrenics/clay nanocomposites. Polymer.

[156] Motamedi P., Bagheri R. (2010). Investigation of the nanostructure and mechanical properties of polypropylene/polyamide 6/layered silicate ternary nanocomposites. Mater. Des..

[157] Huang Z.M., Zhang Y.Z., Kotaki M., Ramakrishna S. (2003). A review on polymer nanofibers by electrospinning and their applications in nanocomposites. Compos. Sci. Technol..

[158] Xue J., Wu T., Dai Y., Xia Y. (2019). Electrospinning and electrospun nanofibers: Methods, materials, and applications. Chem. Rev..

[159] Wang Z., Crandall C., Sahadevan R., Menkhaus T.J., Fong H. (2017). Microfiltration performance of electrospun nanofiber membranes with varied fiber diameters and different membrane porosities and thicknesses. Polymer.

[160] Dolina J., Jiříček T., Lederer T. (2015). Biocide modification of ultrafiltration membranes using nanofiber structures. Desalin. Water Treat..

[161] Wang X., Fang D., Hsiao B.S., Chu B. (2014). Nanofiltration membranes based on thin-film nanofibrous composites. J. Membr. Sci..

[162] Wang X., Ma H., Chu B., Hsiao B.S. (2017). Thin-film nanofibrous composite reverse osmosis membranes for desalination. Desalination.

[163] Li J.J., Zhu L.T., Luo Z.H. (2016). Electrospun fibrous membrane with enhanced swithchable oil/water wettability for oily water separation. Chem. Eng. J..

[164] Najafi M., Frey M.W. (2020). Electrospun nanofibers for chemical separation. Nanomaterials.

[165] Bhardwaj N., Kundu S.C. (2010). Electrospinning: A fascinating fiber fabrication technique. Biotechnol. Adv..

[166] Hou D., Lin D., Ding C., Wang D., Wang J. (2017). Fabrication and characterization of electrospun superhydrophobic PVDF-HFP/SiNPs hybrid membrane for membrane distillation. Sep. Purif. Technol..

[167] Yar A., Haspulat B., Üstün T., Eskizeybek V., Avci A., Kamiş H., Achour S. (2017). Electrospun TiO_2_/ZnO/PAN hybrid nanofiber membranes with efficient photocatalytic activity. RSC Adv..

[168] Essalhi M., Khayet M. (2014). Self-sustained webs of polyvinylidene fluoride electrospun nano-fibers: Effects of polymer concentration and desalination by direct contact membrane distillation. J. Membr. Sci..

[169] Ziabari M., Mottaghitalab V., Haghi A.K. (2009). Application of direct tracking method for measuring electrospun nanofiber diameter. Braz. J. Chem. Eng..

[170] Biswas P., Bandyopadhyaya R. (2017). Biofouling prevention using silver nanoparticle impregnated polyethersulfone (PES) membrane: *E. coli* cell-killing in a continuous cross-flow membrane module. J. Colloid Interface Sci..

[171] Zheng Y., Gong R.H., Zeng Y. (2015). Multijet motion and deviation in electrospinning. RSC Adv..

[172] Li D., Xia Y. (2004). Direct fabrication of composite and ceramic hollow nanofibers by electrospinning. Nano Lett..

[173] Lee G.H., Song J.C., Yoon K.B. (2010). Controlled wall thickness and porosity of polymeric hollow nanofibers by coaxial electrospinning. Macromol. Res..

[174] Li D., Xia Y. (2003). Fabrication of titania nanofibers by electrospinning. Nano Lett..

[175] Malwal D., Gopinath P. (2015). Fabrication and characterization of poly(ethylene oxide) templated nickel oxide nanofibers for dye degradation. Environ. Sci. Nano.

[176] Ray S.S., Chen S.S., Li C.W., Nguyen N.C., Nguyen H.T. (2016). A comprehensive review: Electrospinning technique for fabrication and surface modification of membranes for water treatment application. RSC Adv..

[177] Lee C.G., Javed H., Zhang D., Kim J.H., Westerhoff P., Li Q., Alvarez P.J.J. (2018). Porous Electrospun Fibers Embedding TiO_2_ for Adsorption and Photocatalytic Degradation of Water Pollutants. Environ. Sci. Technol..

[178] Ognibene G., Gangemi C.M.A., D’Urso A., Purrello R., Cicala G., Fragalà M.E. (2018). Combined Approach to Remove and Fast Detect Heavy Metals in Water Based on PES-TiO_2_ Electrospun Mats and Porphyrin Chemosensors. ACS Omega.

[179] Mohamed A., Osman T.A., Toprak M.S., Muhammed M., Yilmaz E., Uheida A. (2016). Visible light photocatalytic reduction of Cr(VI) by surface modified CNT/titanium dioxide composites nanofibers. J. Mol. Catal. A Chem..

[180] Schiffman J.D., Elimelech M. (2011). Antibacterial Activity of Electrospun Polymer Mats with Incorporated Narrow Diameter Single-Walled Carbon Nanotubes. ACS Appl. Mater. Interfaces.

[181] Ford E.N.J., Suthiwangcharoen N., D’Angelo P.A., Nagarajan R. (2014). Role of single-walled carbon nanotubes on ester hydrolysis and topography of electrospun bovine serum albumin/poly(vinyl alcohol) membranes. ACS Appl. Mater. Interfaces.

[182] Liao Y., Loh C.H., Wang R., Fane A.G. (2014). Electrospun superhydrophobic membranes with unique structures for membrane distillation. ACS Appl. Mater. Interfaces.

[183] Obaid M., Ghouri Z.K., Fadali O.A., Khalil K.A., Almajid A.A., Barakat N.A.M. (2016). Amorphous SiO_2_ NP-Incorporated Poly(vinylidene fluoride) Electrospun Nanofiber Membrane for High Flux Forward Osmosis Desalination. ACS Appl. Mater. Interfaces.

[184] Li X., Yu X., Cheng C., Deng L., Wang M., Wang X. (2015). Electrospun Superhydrophobic Organic/Inorganic Composite Nanofibrous Membranes for Membrane Distillation. ACS Appl. Mater. Interfaces.

[185] Son W.K., Youk J.H., Park W.H. (2006). Antimicrobial cellulose acetate nanofibers containing silver nanoparticles. Carbohydr. Polym..

[186] De Faria A.F., Perreault F., Shaulsky E., Arias Chavez L.H., Elimelech M. (2015). Antimicrobial Electrospun Biopolymer Nanofiber Mats Functionalized with Graphene Oxide-Silver Nanocomposites. ACS Appl. Mater. Interfaces.

[187] Karagoz S., Kiremitler N.B., Sakir M., Salem S., Onses M.S., Sahmetlioglu E., Ceylan A., Yilmaz E. (2020). Synthesis of Ag and TiO_2_ modified polycaprolactone electrospun nanofibers (PCL/TiO_2_-Ag NFs) as a multifunctional material for SERS, photocatalysis and antibacterial applications. Ecotoxicol. Environ. Saf..

[188] Kayaci F., Ozgit-Akgun C., Donmez I., Biyikli N., Uyar T. (2012). Polymer-inorganic core-shell nanofibers by electrospinning and atomic layer deposition: Flexible nylon-ZnO core-shell nanofiber mats and their photocatalytic activity. ACS Appl. Mater. Interfaces.

[189] Kim J.H., Joshi M.K., Lee J., Park C.H., Kim C.S. (2018). Polydopamine-assisted immobilization of hierarchical zinc oxide nanostructures on electrospun nanofibrous membrane for photocatalysis and antimicrobial activity. J. Colloid Interface Sci..

[190] Barhoum A., Bechelany M., Makhlouf A.S.H. (2019). Handbook of Nanofibers.

[191] Xiao S., Shen M., Guo R., Wang S., Shi X. (2009). Immobilization of Zerovalent Iron Nanoparticles into Electrospun Polymer Nanofibers: Synthesis, Characterization, and Potential Environmental Applications. J. Phys. Chem. C.

[192] Zhang D., Jin X.Z., Huang T., Zhang N., Qi X.D., Yang J.H., Zhou Z.W., Wang Y. (2019). Electrospun Fibrous Membranes with Dual-Scaled Porous Structure: Super Hydrophobicity, Super Lipophilicity, Excellent Water Adhesion, and Anti-Icing for Highly Efficient Oil Adsorption/Separation. ACS Appl. Mater. Interfaces.

[193] Letnik I., Avrahami R., Rokem J.S., Greiner A., Zussman E., Greenblatt C. (2015). Living Composites of Electrospun Yeast Cells for Bioremediation and Ethanol Production. Biomacromolecules.

[194] Yap C.Y., Chua C.K., Dong Z.L., Liu Z.H., Zhang D.Q., Loh L.E., Sing S.L. (2015). Review of selective laser melting: Materials and applications. Appl. Phys. Rev..

[195] Fasel U., Keidel D., Baumann L., Cavolina G., Eichenhofer M., Ermanni P. (2020). Composite additive manufacturing of morphing aerospace structures. Manuf. Lett..

[196] Lewandowski J.J., Seifi M. (2016). Metal Additive Manufacturing: A Review of Mechanical Properties. Annu. Rev. Mater. Res..

[197] Schmitt M., Mehta R.M., Kim I.Y. (2020). Additive manufacturing infill optimization for automotive 3D-printed ABS components. Rapid Prototyp. J..

[198] Lim C.W.J., Le K.Q., Lu Q., Wong C.H. (2016). An Overview of 3-D Printing in Manufacturing, Aerospace, and Automotive Industries. IEEE Potentials.

[199] Tay Y.W.D., Panda B., Paul S.C., Noor Mohamed N.A., Tan M.J., Leong K.F. (2017). 3D printing trends in building and construction industry: A review. Virtual Phys. Prototyp..

[200] Lin K., Zhang D., Macedo M.H., Cui W., Sarmento B., Shen G. (2019). Advanced Collagen-Based Biomaterials for Regenerative Biomedicine. Adv. Funct. Mater..

[201] Derakhshanfar S., Mbeleck R., Xu K., Zhang X., Zhong W., Xing M. (2018). 3D bioprinting for biomedical devices and tissue engineering: A review of recent trends and advances. Bioact. Mater..

[202] Sun J., Zhou W., Huang D., Fuh J.Y.H., Hong G.S. (2015). An Overview of 3D Printing Technologies for Food Fabrication. Food Bioprocess Technol..

[203] Lalia B.S., Kochkodan V., Hashaikeh R., Hilal N. (2013). A review on membrane fabrication: Structure, properties and performance relationship. Desalination.

[204] Issac M.N., Kandasubramanian B. (2020). Review of manufacturing three-dimensional-printed membranes for water treatment. Environ. Sci. Pollut. Res..

[205] Ngo T.D., Kashani A., Imbalzano G., Nguyen K.T.Q., Hui D. (2018). Additive manufacturing (3D printing): A review of materials, methods, applications and challenges. Compos. Part B Eng..

[206] Low Z.X., Chua Y.T., Ray B.M., Mattia D., Metcalfe I.S., Patterson D.A. (2017). Perspective on 3D printing of separation membranes and comparison to related unconventional fabrication techniques. J. Membr. Sci..

[207] Yusuf A., Sodiq A., Giwa A., Eke J., Pikuda O., De Luca G., Di Salvo J.L., Chakraborty S. (2020). A review of emerging trends in membrane science and technology for sustainable water treatment. J. Clean. Prod..

[208] Tijing L.D., Dizon J.R.C., Ibrahim I., Nisay A.R.N., Shon H.K., Advincula R.C. (2020). 3D printing for membrane separation, desalination and water treatment. Appl. Mater. Today.

[209] Koh J.J., Lim G.J.H., Zhou X., Zhang X., Ding J., He C. (2019). 3D-Printed Anti-Fouling Cellulose Mesh for Highly Efficient Oil/Water Separation Applications. ACS Appl. Mater. Interfaces.

[210] Sangiorgi A., Gonzalez Z., Ferrandez-Montero A., Yus J., Sanchez-Herencia A.J., Galassi C., Sanson A., Ferrari B. (2019). 3D Printing of Photocatalytic Filters Using a Biopolymer to Immobilize TiO_2_ Nanoparticles. J. Electrochem. Soc..

[211] Gude V.G. (2018). Emerging Technologies for Sustainable Desalination Handbook.

[212] Kononova S.V., Gubanova G.N., Korytkova E.N., Sapegin D.A., Setnickova K., Petrychkovych R., Uchytil P. (2018). Polymer nanocomposite membranes. Appl. Sci..

[213] Liang C.Z., Chung T.S., Lai J.Y. (2019). A review of polymeric composite membranes for gas separation and energy production. Prog. Polym. Sci..

[214] Bassyouni M., Abdel-Aziz M.H., Zoromba M.S., Abdel-Hamid S.M.S., Drioli E. (2019). A review of polymeric nanocomposite membranes for water purification. J. Ind. Eng. Chem..

[215] Li N.N., Long R.B., Henley E.J. (1965). Membrane Separation Processes.

[216] Palencia M. (2019). Fundamental and Methodological Aspects of Porous Membrane Characterization by Hydrodynamic Permeability Test-A review. J. Sci. Technol. Appl..

[217] Palencia M. (2015). Liquid-phase polymer-based retention: Theory, modeling, and application for the removal of pollutant inorganic ions. J. Chem..

[218] Ching C.B., Hidajat K., Uddin M.S. (1989). Evaluation of Equilibrium and Kinetic Parameters of Smaller Molecular Size Amino Acids on KX Zeolite Crystals via Liquid Chromatographic Techniques. Sep. Sci. Technol..

[219] Lerma T.A., Martínez G., Palencia M. (2017). Generation of thiolated porous surfaces by interpenetrating polymeric networks: Study of their surface properties. J. Sci. Technol. Appl..

[220] Palencia M.S., Berrio M.E., Palencia S.L. (2017). Effect of capping agent and diffusivity of different silver nanoparticles on their antibacterial properties. J. Nanosci. Nanotechnol..

[221] Ambrosio R., Carrillo A., Mota M.L., de la Torre K., Torrealba R., Moreno M., Vazquez H., Flores J., Vivaldo I. (2018). Polymeric nanocomposites membranes with high permittivity based on PVA-ZnO nanoparticles for potential applications in flexible electronics. Polymers.

[222] Nizamuddin S., Maryam S., Baloch H.A., Siddiqui M.T.H., Takkalkar P., Mubarak N.M., Jatoi A.S., Abbasi S.A., Griffin G.J., Qureshi K., Inamuddin, Thomas S., Kumar Mishra R., Asiri A.M. (2019). Electrical properties of sustainable nano-composites containing nano-fillers: Dielectric properties and electrical conductivity. Sustainable Polymer Composites and Nanocomposites.

[223] Drioli E., Giorno L. (2010). Comprehensive Membrane Science and Engineering.

[224] Rostamzadeh H., Namin A.S., Ghaebi H., Amidpour M. (2018). Performance assessment and optimization of a humidification dehumidification (HDH) system driven by absorption-compression heat pump cycle. Desalination.

[225] Som C., Berges M., Chaudhry Q., Dusinska M., Fernandes T.F., Olsen S.I., Nowack B. (2010). The importance of life cycle concepts for the development of safe nanoproducts. Toxicology.

[226] Zodrow K., Brunet L., Mahendra S., Li D., Zhang A., Li Q., Alvarez P.J.J. (2009). Polysulfone ultrafiltration membranes impregnated with silver nanoparticles show improved biofouling resistance and virus removal. Water Res..

[227] Kingston C., Zepp R., Andrady A., Boverhof D., Fehir R., Hawkins D., Roberts J., Sayre P., Shelton B., Sultan Y. (2014). Release characteristics of selected carbon nanotube polymer composites. Carbon N. Y..

[228] Vilar G., Fernández-Rosas E., Puntes V., Jamier V., Aubouy L., Vázquez-Campos S. (2013). Monitoring migration and transformation of nanomaterials in polymeric composites during accelerated aging. J. Phys. Conf. Ser..

[229] Hu M., Zhong K., Liang Y., Ehrman S.H., Mi B. (2017). Effects of Particle Morphology on the Antibiofouling Performance of Silver Embedded Polysulfone Membranes and Rate of Silver Leaching. Ind. Eng. Chem. Res..

[230] Hu R., He Y., Zhang C., Zhang R., Li J., Zhu H. (2017). Graphene oxide-embedded polyamide nanofiltration membranes for selective ion separation. J. Mater. Chem. A.

[231] Li X., Fang X., Pang R., Li J., Sun X., Shen J., Han W., Wang L. (2014). Self-assembly of TiO_2_ nanoparticles around the pores of PES ultrafiltration membrane for mitigating organic fouling. J. Membr. Sci..

[232] Wan H., Briot N.J., Saad A., Ormsbee L., Bhattacharyya D. (2017). Pore functionalized PVDF membranes with in-situ synthesized metal nanoparticles: Material characterization, and toxic organic degradation. J. Membr. Sci..

[233] Lin Y.H., Tseng H.H., Wey M.Y., Lin M. (2009). Der Characteristics, morphology, and stabilization mechanism of PAA250K-stabilized bimetal nanoparticles. Colloids Surf. A Physicochem. Eng. Asp..

[234] Wu J., Yu C., Li Q. (2015). Regenerable antimicrobial activity in polyamide thin film nanocomposite membranes. J. Membr. Sci..

[235] Salas E.C., Sun Z., Lüttge A., Tour J.M. (2010). Reduction of Graphene Oxide via Bacterial Respiration. ACS Nano.

[236] Wang G., Qian F., Saltikov C.W., Jiao Y., Li Y. (2011). Microbial reduction of graphene oxide by Shewanella. Nano Res..

[237] Akhavan O., Ghaderi E. (2012). Escherichia coli bacteria reduce graphene oxide to bactericidal graphene in a self-limiting manner. Carbon N. Y..

[238] Liu J., Gao Y., Cao D., Zhang L., Guo Z. (2011). Nanoparticle dispersion and aggregation in polymer nanocomposites: Insights from molecular dynamics simulation. Langmuir.

[239] Yuan Y., Lee T.R. (2013). Contact angle and wetting properties. Surface Science Techniques.

[240] Kim S.H., Kwak S.Y., Sohn B.H., Park T.H. (2003). Design of TiO_2_ nanoparticle self-assembled aromatic polyamide thin-film-composite (TFC) membrane as an approach to solve biofouling problem. J. Membr. Sci..

[241] Kwak S.Y., Kim S.H., Kim S.S. (2001). Hybrid organic/inorganic reverse osmosis (RO) membrane for bactericidal anti-fouling. 1. Preparation and characterization of TiO_2_ nanoparticle self-assembled aromatic polyamide thin-film-composite (TFC) membrane. Environ. Sci. Technol..

[242] Han Y., Xu Z., Gao C. (2013). Ultrathin graphene nanofiltration membrane for water purification. Adv. Funct. Mater..

[243] Brady-Estévez A.S., Kang S., Elimelech M. (2008). A single-walled-carbon-nanotube filter for removal of viral and bacterial pathogens. Small.

[244] Bae T.H., Kim I.C., Tak T.M. (2006). Preparation and characterization of fouling-resistant TiO_2_ self-assembled nanocomposite membranes. J. Membr. Sci..

[245] Luo M.L., Zhao J.Q., Tang W., Pu C.S. (2005). Hydrophilic modification of poly(ether sulfone) ultrafiltration membrane surface by self-assembly of TiO_2_ nanoparticles. Appl. Surf. Sci..

[246] Li J.H., Xu Y.Y., Zhu L.P., Wang J.H., Du C.H. (2009). Fabrication and characterization of a novel TiO_2_ nanoparticle self-assembly membrane with improved fouling resistance. J. Membr. Sci..

[247] Yang S., Gu J.S., Yu H.Y., Zhou J., Li S.F., Wu X.M., Wang L. (2011). Polypropylene membrane surface modification by RAFT grafting polymerization and TiO_2_ photocatalysts immobilization for phenol decomposition in a photocatalytic membrane reactor. Sep. Purif. Technol..

[248] Madaeni S.S., Ghaemi N., Alizadeh A., Joshaghani M. (2011). Influence of photo-induced superhydrophilicity of titanium dioxide nanoparticles on the anti-fouling performance of ultrafiltration membranes. Appl. Surf. Sci..

[249] Fan Z., Wang Z., Sun N., Wang J., Wang S. (2008). Performance improvement of polysulfone ultrafiltration membrane by blending with polyaniline nanofibers. J. Membr. Sci..

[250] Tiraferri A., Kang Y., Giannelis E.P., Elimelech M. (2012). Superhydrophilic thin-film composite forward osmosis membranes for organic fouling control: Fouling behavior and antifouling mechanisms. Environ. Sci. Technol..

[251] Wang Z., Ma H., Hsiao B.S., Chu B. (2014). Nanofibrous ultrafiltration membranes containing cross-linked poly(ethylene glycol) and cellulose nanofiber composite barrier layer. Polymer.

[252] Rasheed I.A., Rehan Z.A., Khalid T., Zahid M., Ahmad H., Sadrzadeh M., Mohammadi T. (2019). Prospects of nanocomposite membranes in commercial scale. Nanocomposite Membranes for Water and Gas Separation.

[253] Zahid M., Rashid A., Akram S., Rehan Z.A., Razzaq W. (2018). A Comprehensive Review on Polymeric Nano-Composite Membranes for Water Treatment. J. Membr. Sci. Technol..

[254] Tiraferri A., Vecitis C.D., Elimelech M. (2011). Covalent binding of single-walled carbon nanotubes to polyamide membranes for antimicrobial surface properties. ACS Appl. Mater. Interfaces.

[255] Kang G.-D., Cao Y.-M. (2012). Development of antifouling reverse osmosis membranes for water treatment: A review. Water Res..

[256] Yu H., Zhang X., Zhang Y., Liu J., Zhang H. (2013). Development of a hydrophilic PES ultrafiltration membrane containing SiO_2_@N-Halamine nanoparticles with both organic antifouling and antibacterial properties. Desalination.

[257] Chkirida S., Zari N., Achour R., Hassoune H., Lachehab A., Qaiss A.e.k., Bouhfid R. (2021). Highly synergic adsorption/photocatalytic efficiency of Alginate/Bentonite impregnated TiO2 beads for wastewater treatment. J. Photochem. Photobiol. A Chem..

[258] Bilal M., Ihsanullah I., Younas M., Ul Hassan Shah M. (2022). Recent advances in applications of low-cost adsorbents for the removal of heavy metals from water: A critical review. Sep. Purif. Technol..

[259] Pang Y., Yu J., Tang L., Zeng G., Zhu C., Wei X., Tang L., Deng Y., Wang J., Wang J., Zeng G. (2018). Magnetic Nanohybrid Materials for Water-Pollutant Removal. Nanohybrid and Nanoporous Materials for Aquatic Pollution Control.

[260] Fan C., Li K., Wang Y., Qian X., Jia J. (2016). The stability of magnetic chitosan beads in the adsorption of Cu^2+^. RSC Adv..

[261] Salvador F., Martin-Sanchez N., Sanchez-Montero M.J., Montero J., Izquierdo C. (2013). Regeneration of activated carbons contaminated by phenol using supercritical water. J. Supercrit. Fluids.

[262] Sun Z., Li C., Wu D. (2010). Removal of methylene blue from aqueous solution by adsorption onto zeolite synthesized from coal fly ash and its thermal regeneration. J. Chem. Technol. Biotechnol..

[263] Shah I.K., Pre P., Alappat B.J. (2013). Steam Regeneration of Adsorbents: An Experimental and Technical Review. Chem. Sci. Trans..

[264] Chkirida S., Zari N., Bouhfid R., Qaiss A.e.k. (2021). Insight into the bionanocomposite applications on wastewater decontamination: Review. J. Water Process Eng..

[265] Hlongwane G.N., Sekoai P.T., Meyyappan M., Moothi K. (2019). Simultaneous removal of pollutants from water using nanoparticles: A shift from single pollutant control to multiple pollutant control. Sci. Total Environ..

[266] Wu Z., Deng W., Zhou W., Luo J. (2019). Novel magnetic polysaccharide/graphene oxide @Fe_3_O_4_ gel beads for adsorbing heavy metal ions. Carbohydr. Polym..

[267] Shchipunov Y. (2012). Bionanocomposites: Green sustainable materials for the near future. Pure Appl. Chem..

[268] Darder M., Aranda P., Ferrer M.L., Gutiérrez M.C., Del Monte F., Ruiz-Hitzky E. (2011). Progress in bionanocomposite and bioinspired foams. Adv. Mater..

[269] Gupta O., Roy S., Sadrzadeh M., Mohammadi T. (2019). Recent progress in the development of nanocomposite membranes. Nanocomposite Membranes for Water and Gas Separation.

[270] Baten R., Stummeyer K. (2013). How sustainable can desalination be?. Desalin. Water Treat..

[271] Miller S., Shemer H., Semiat R. (2015). Energy and environmental issues in desalination. Desalination.

[272] Haddad B.M. (2013). A case for an ecological-economic research program for desalination. Desalination.

[273] Beer C., Foldbjerg R., Hayashi Y., Sutherland D.S., Autrup H. (2012). Toxicity of silver nanoparticles-Nanoparticle or silver ion?. Toxicol. Lett..

[274] Naseri R., Davoodi R. Commercialization of Nanotechnology in Developing Countries. Proceedings of the 3rd International Conference on Information and Financial Engineering (ICIFE 2011).

[275] Mazumder S., Sarkar D., Puri I.K. (2014). Nanotechnology Commercialization: Prospects in India. J. Mater. Sci. Nanotechnol..

[276] Aithal P.S., Aithal S. (2020). Nanotechnological innovations & business environment for Indian automobile sector: A futuristic approach. J. Sci. Res. Mod..

[277] Aithal S. (2016). Nanotechnology Innovations & Business Opportunities: A Review. Int. J. Manag. IT Eng..

[278] Karthikeyan P., Vigneshwaran S., Meenakshi S. (2020). Al^3+^ incorporated chitosan-gelatin hybrid microspheres and their use for toxic ions removal: Assessment of its sustainability metrics. Environ. Chem. Ecotoxicol..

[279] Buzea C., Pacheco I.I., Robbie K. (2007). Nanomaterials and nanoparticles: Sources and toxicity. Biointerphases.

[280] Pacheco I., Buzea C., Kharissova O.V., Torres-Martínez L.M., Kharisov B.I. (2021). Nanomaterials and Nanocomposites: Classification and Toxicity. Handbook of Nanomaterials and Nanocomposites for Energy and Environmental Applications.

[281] Sarin H. (2010). Physiologic upper limits of pore size of different blood capillary types and another perspective on the dual pore theory of microvascular permeability. J. Angiogenes. Res..

[282] Wu T., Tang M. (2018). Review of the effects of manufactured nanoparticles on mammalian target organs. J. Appl. Toxicol..

[283] Karmakar A., Zhang Q., Zhang Y. (2014). Neurotoxicity of nanoscale materials. J. Food Drug Anal..

[284] Schrand A.M., Rahman M.F., Hussain S.M., Schlager J.J., Smith D.A., Syed A.F. (2010). Metal-based nanoparticles and their toxicity assessment. Wiley Interdiscip. Rev. Nanomed. Nanobiotechnol..

[285] Castranova V., Schulte P.A., Zumwalde R.D. (2013). Occupational nanosafety considerations for carbon nanotubes and carbon nanofibers. Acc. Chem. Res..

[286] Dahm M.M., Schubauer-Berigan M.K., Evans D.E., Birch M.E., Fernback J.E., Deddens J.A. (2015). Carbon nanotube and nanofiber exposure assessments: An analysis of 14 site visits. Ann. Occup. Hyg..

[287] Ding Y., Kuhlbusch T.A.J., Van Tongeren M., Jiménez A.S., Tuinman I., Chen R., Alvarez I.L., Mikolajczyk U., Nickel C., Meyer J. (2017). Airborne engineered nanomaterials in the workplace—A review of release and worker exposure during nanomaterial production and handling processes. J. Hazard. Mater..

[288] Kuijpers E., Bekker C., Brouwer D., Le Feber M., Fransman W. (2017). Understanding workers’ exposure: Systematic review and data-analysis of emission potential for NOAA. J. Occup. Environ. Hyg..

[289] Liu Z., Zhang F.S. (2010). Nano-zerovalent iron contained porous carbons developed from waste biomass for the adsorption and dechlorination of PCBs. Bioresour. Technol..

[290] Varanasi P., Fullana A., Sidhu S. (2007). Remediation of PCB contaminated soils using iron nano-particles. Chemosphere.

[291] Xu X., Wang Q., Choi H.C., Kim Y.H. (2010). Encapsulation of iron nanoparticles with PVP nanofibrous membranes to maintain their catalytic activity. J. Membr. Sci..

[292] Tong M., Yuan S., Long H., Zheng M., Wang L., Chen J. (2011). Reduction of nitrobenzene in groundwater by iron nanoparticles immobilized in PEG/nylon membrane. J. Contam. Hydrol..

[293] Dong T., Luo H., Wang Y., Hu B., Chen H. (2011). Stabilization of Fe-Pd bimetallic nanoparticles with sodium carboxymethyl cellulose for catalytic reduction of para-nitrochlorobenzene in water. Desalination.

[294] Konstantinou I.K., Albanis T.A. (2004). TiO_2_-assisted photocatalytic degradation of azo dyes in aqueous solution: Kinetic and mechanistic investigations: A review. Appl. Catal. B Environ..

[295] Zhu H., Jiang R., Xiao L., Chang Y., Guan Y., Li X., Zeng G. (2009). Photocatalytic decolorization and degradation of Congo Red on innovative crosslinked chitosan/nano-CdS composite catalyst under visible light irradiation. J. Hazard. Mater..

[296] Konstantinou I.K., Sakellarides T.M., Sakkas V.A., Albanis T.A. (2001). Photocatalytic degradation of selected s-triazine herbicides and oganophosphorus insecticides over aqueous TiO_2_ suspensions. Environ. Sci. Technol..

[297] Song H., Carraway E.R., Kim Y.H., Batchelor B., Jeon B.H., Kim J.G. (2008). Amendment of hydroxyapatite in reduction of tetrachloroethylene by zero-valent zinc: Its rate enhancing effect and removal of Zn(II). Chemosphere.

[298] Wang Z., Huang W., Fennell D.E., Peng P. (2008). Kinetics of reductive dechlorination of 1,2,3,4-TCDD in the presence of zero-valent zinc. Chemosphere.

[299] Mahendra S., Li Q., Lyon D.Y., Brunet L., Alvarez P.J.J., Savage N., Diallo M., Duncan J., Street A., Sustich R. (2009). Nanotechnology-Enabled Water Disinfection and Microbial Control: Merits and Limitations. Nanotechnology Applications for Clean Water.

[300] Li Q., Mahendra S., Lyon D.Y., Brunet L., Liga M.V., Li D., Alvarez P.J.J. (2008). Antimicrobial nanomaterials for water disinfection and microbial control: Potential applications and implications. Water Res..

[301] Jain P., Pradeep T. (2005). Potential of silver nanoparticle-coated polyurethane foam as an antibacterial water filter. Biotechnol. Bioeng..

[302] Lv Y., Liu H., Wang Z., Liu S., Hao L., Sang Y., Liu D., Wang J., Boughton R.I. (2009). Silver nanoparticle-decorated porous ceramic composite for water treatment. J. Membr. Sci..

[303] Dankovich T.A., Gray D.G. (2011). Bactericidal paper impregnated with silver nanoparticles for point-of-use water treatment. Environ. Sci. Technol..

[304] Abebe L.S., Smith J.A., Narkiewicz S., Oyanedel-Craver V., Conaway M., Singo A., Amidou S., Mojapelo P., Brant J., Dillingham R. (2014). Ceramic water filters impregnated with silver nanoparticles as a point-of-use water-treatment intervention for HIV-positive individuals in Limpopo Province, South Africa: A pilot study of technological performance and human health benefits. J. Water Health.

[305] Li Y., Bhalli J.A., Ding W., Yan J., Pearce M.G., Sadiq R., Cunningham C.K., Jones M.Y., Monroe W.A., Howard P.C. (2014). Cytotoxicity and genotoxicity assessment of silver nanoparticles in mouse. Nanotoxicology.

[306] Carlson C., Hussein S.M., Schrand A.M., Braydich-Stolle L.K., Hess K.L., Jones R.L., Schlager J.J. (2008). Unique cellular interaction of silver nanoparticles: Size-dependent generation of reactive oxygen species. J. Phys. Chem. B.

[307] Trouiller B., Reliene R., Westbrook A., Solaimani P., Schiestl R.H. (2009). Titanium dioxide nanoparticles induce DNA damage and genetic instability in vivo in mice. Cancer Res..

[308] Park E.J., Bae E., Yi J., Kim Y., Choi K., Lee S.H., Yoon J., Lee B.C., Park K. (2010). Repeated-dose toxicity and inflammatory responses in mice by oral administration of silver nanoparticles. Environ. Toxicol. Pharmacol..

[309] Kim Y.S., Kim J.S., Cho H.S., Rha D.S., Kim J.M., Park J.D., Choi B.S., Lim R., Chang H.K., Chung Y.H. (2008). Twenty-eight-day oral toxicity, genotoxicity, and gender-related tissue distribution of silver nanoparticles in Sprague-Dawley rats. Inhal. Toxicol..

[310] Adibzadeh S., Bazgir S., Katbab A.A. (2014). Fabrication and characterization of chitosan/poly(vinyl alcohol) electrospun nanofibrous membranes containing silver nanoparticles for antibacterial water filtration. Iran. Polym. J..

[311] Westerhoff P., Atkinson A., Fortner J., Wong M.S., Zimmerman J., Gardea-Torresdey J., Ranville J., Herckes P. (2018). Low risk posed by engineered and incidental nanoparticles in drinking water. Nat. Nanotechnol..

[312] Chkirida S., Zari N., Qaiss A.E.K., Bouhfid R., Prasad R., Karchiyappan T. (2019). Nanocomposite Materials Based on TiO_2_/Clay for Wastewater Treatment. Nanotechnology in the Life Sciences.

[313] Bundschuh M., Filser J., Lüderwald S., McKee M.S., Metreveli G., Schaumann G.E., Schulz R., Wagner S. (2018). Nanoparticles in the environment: Where do we come from, where do we go to?. Environ. Sci. Eur..

[314] Jabbari V., Veleta J.M., Zarei-Chaleshtori M., Gardea-Torresdey J., Villagrán D. (2016). Green synthesis of magnetic MOF@GO and MOF@CNT hybrid nanocomposites with high adsorption capacity towards organic pollutants. Chem. Eng. J..

[315] Abdi J., Vossoughi M., Mahmoodi N.M., Alemzadeh I. (2017). Synthesis of metal-organic framework hybrid nanocomposites based on GO and CNT with high adsorption capacity for dye removal. Chem. Eng. J..

[316] Torad N.L., Hu M., Ishihara S., Sukegawa H., Belik A.A., Imura M., Ariga K., Sakka Y., Yamauchi Y. (2014). Direct synthesis of MOF-derived nanoporous carbon with magnetic Co nanoparticles toward efficient water treatment. Small.

